# Systems Perturbation Analysis of a Large-Scale Signal Transduction Model Reveals Potentially Influential Candidates for Cancer Therapeutics

**DOI:** 10.3389/fbioe.2016.00010

**Published:** 2016-02-11

**Authors:** Bhanwar Lal Puniya, Laura Allen, Colleen Hochfelder, Mahbubul Majumder, Tomáš Helikar

**Affiliations:** ^1^Department of Biochemistry, University of Nebraska-Lincoln, Lincoln, NE, USA; ^2^Department of Mathematics, University of Nebraska at Omaha, Omaha, NE, USA; ^3^Albert Einstein College of Medicine, New York, NY, USA

**Keywords:** computational modeling, *in silico* perturbation analysis, signal transduction, cancer, therapeutic targets

## Abstract

Dysregulation in signal transduction pathways can lead to a variety of complex disorders, including cancer. Computational approaches such as network analysis are important tools to understand system dynamics as well as to identify critical components that could be further explored as therapeutic targets. Here, we performed perturbation analysis of a large-scale signal transduction model in extracellular environments that stimulate cell death, growth, motility, and quiescence. Each of the model’s components was perturbed under both loss-of-function and gain-of-function mutations. Using 1,300 simulations under both types of perturbations across various extracellular conditions, we identified the most and least influential components based on the magnitude of their influence on the rest of the system. Based on the premise that the most influential components might serve as better drug targets, we characterized them for biological functions, housekeeping genes, essential genes, and druggable proteins. The most influential components under all environmental conditions were enriched with several biological processes. The inositol pathway was found as most influential under inactivating perturbations, whereas the kinase and small lung cancer pathways were identified as the most influential under activating perturbations. The most influential components were enriched with essential genes and druggable proteins. Moreover, known cancer drug targets were also classified in influential components based on the affected components in the network. Additionally, the systemic perturbation analysis of the model revealed a network motif of most influential components which affect each other. Furthermore, our analysis predicted novel combinations of cancer drug targets with various effects on other most influential components. We found that the combinatorial perturbation consisting of PI3K inactivation and overactivation of IP3R1 can lead to increased activity levels of apoptosis-related components and tumor-suppressor genes, suggesting that this combinatorial perturbation may lead to a better target for decreasing cell proliferation and inducing apoptosis. Finally, our approach shows a potential to identify and prioritize therapeutic targets through systemic perturbation analysis of large-scale computational models of signal transduction. Although some components of the presented computational results have been validated against independent gene expression data sets, more laboratory experiments are warranted to more comprehensively validate the presented results.

## Introduction

Recent advances in systems biology and computational biology have introduced methods for the visualization, comprehension, and interpretation of big data in biomedical research. These fields provide an array of methodologies including computer simulations that can be used to generate new hypotheses and identify which hypotheses might be more productive to undertake experimentally, and eliminate hypotheses with little chance of success (Kitano, 2002a,b; Ghosh et al., 2011). These methods can be effective in navigating complex network problems associated with diseases. Many diseases and pathologies can be characterized by the dysregulation or dysfunction of multiple molecular components that are connected within these highly intertwined biological and biochemical networks (Loscalzo and Barabasi, 2011). Biological networks, including biochemical signal transduction networks, consist of a large number of highly interconnected pathways that give rise to complex, non-linear dynamics governing various cellular functions (Helikar et al., 2008; Helikar and Rogers, 2009). Disruptions of these networks, such as mutations or disease states can have drastic effects upon the whole system. These effects are difficult to predict from static network diagrams.

However, understanding the hierarchy of these changes remains a paramount problem. Often the specific causal interactions of the disease state are hidden within the massive cell-wide alterations, making attempts to reverse a disease state more challenging. In addition, the specific causal interactions are difficult to predict making the development of a potential therapeutic target results in unforeseen side effects (Singh and Singh, 2012). The unwanted effects of these drugs are often drastic as seen with many cancer medications (Kayl and Meyers, 2006; Lotfi-Jam et al., 2008; Singh and Singh, 2012). These challenges are further exacerbated by drug resistance that can render therapies ineffective. Therefore, it is necessary to gain a systems level understanding of the components associated with the disease states.

In recent years, targeted therapy has been used for multiple diseases, e.g., cancer (Vanneman and Dranoff, 2012), and often involve the activation or inactivation of a specific component in a biological network by a small molecule or drug, for instance. Perturbation analyses allow one to interrogate the structure and dynamic footprint of the underlying biological system. Perturbation biology has been proposed as an approach to reduce the collateral damage caused by non-specific drugs. Computational network perturbations and new methods to evaluate the robustness of a given network can help identify more effective network components to target in order to obtain desired outcomes with minimal disruption to the rest of the network (Molinelli et al., 2013).

In order to fully leverage the potential of computational network perturbation analyses large dynamical models are necessary. A wide spectrum of modeling approaches exists ranging from detailed (but less scalable) differential equation-based systems to large (but not dynamic) static networks. In the middle are approaches such as logical modeling that are relatively scalable while capable of capturing the dynamic nature of biological systems (Le Novère, 2015). Logical networks, namely Boolean networks, have been used to describe and simulate a wide spectrum of biological systems ranging in size as well as contextual application (Naldi et al., 2010; Helikar et al., 2012; Madrahimov et al., 2013; Rocha et al., 2013; Conroy et al., 2014). Thus, applying perturbation analysis to large-scale logical models may provide new insights into the system, which could be used to identify novel therapeutic targets.

Herein, we present results from a system-wide perturbation analysis of a large-scale Boolean model of a signal transduction network widely present in many types of cells. Specifically, the model previously described in Helikar et al. (2008) represents signaling events within the integrated epidermal growth factor (EGF), G protein-coupled receptor, and integrin signaling network. The model consists of 137 components (mostly proteins) and 557 biochemical interactions. The simulation-based, system-wide perturbation analyses enabled us to identify the most and least influential components (ones with the most and least impact on the rest of the network). To explore the role and effects of these perturbations in the context of the complex extracellular environment, the simulations and analyses were conducted under four biologically relevant environmental conditions known to stimulate cell growth, cell death, motility, and quiescence (in addition to a set of randomly generated environmental stimuli). In order to investigate potential therapeutic targets, we performed functional annotation and analysis of the most influential signal transduction components under both inactivating (e.g., knockout) and activating (e.g., overexpression) perturbations. The most influential components were found to be enriched with many biological processes and druggable targets. Also, the most influential components under activating perturbations were enriched with more essential genes than the least influential components. We used the most influential components and their upstream regulators to identify novel interactions. We also identified a network of the most influential components consisting of drug targets considered in multiple cancer types. The highest ranked among the most influential components were already explored as drug targets against cancer, including EGFR, PI3K, Raf, Ras, and Erk. Because some of these targets have been reported to be associated with drug resistance (Holohan et al., 2013; Rodon et al., 2013; Wagle et al., 2014), we analyzed additional components of the signal transduction network that could potentially complement drug-resistant targets. As a result of the systemic analysis, we identified one novel combinatorial target, PI3K–IP3R1, with consistent occurrence in all simulated environmental conditions. This combination could be used to suppress cell proliferation while increasing the rate of apoptosis. We simulated the effect of combinatorial perturbation and the results were correlated with the literature, further supporting our predictions.

## Materials and Methods

### Computational Model

The computational model analyzed in this work is a Boolean model of signal transduction in a generic cell type. In Boolean models, each component can assume an active (1) or inactive (0) state at any time *t*. The activity state of the model’s internal components is determined by the regulatory mechanisms of other directly interacting components. These regulatory mechanisms are described with Boolean functions (in the form of truth tables or Boolean expressions). To represent the milieu of stimuli in the extracellular environment, the model contains external components that represent various ligands. The activity level of these components is specified as a probability to simulate different levels of concentrations. This methodology was previously detailed and exemplified in Helikar et al. (2008, 2012), Helikar and Rogers (2009), and Todd and Helikar (2012).

The signal transduction model, previously detailed in Helikar et al. (2008), was constructed manually from around 500 published papers. The model consists of several main signaling pathways, including the receptor tyrosine kinase (EGF receptor), G protein-coupled receptors (G-alpha *i*, G-alpha *q*, G-alpha *s*, and G-alpha 12/13), and the integrin signaling pathways. Each of the 130 components in the model corresponds to a signaling molecule (mainly protein). The model also contains nine external components that represent the extracellular environment (mostly composed of receptor ligands). These external components include the EGF, extracellular matrix (ECM), calcium pump, interleukin 1, and tumor necrosis factor (TNF), ligands for four types of G protein-coupled receptors (αi, αq, αs, and 12/13), and a general stress signal. The final model consists of 137 components (130 internal and 7 external) connected with 557 interactions. The model is fully annotated and freely available *via* the Cell Collective software (Helikar et al., 2012, 2013) at www.thecellcollective.org (under Published Models). Cell Collective, an interactive modeling environment, can be used to download the model (and other logical models published by the community) in several file formats (SBML qual, text file of logical functions, truth tables, etc.), as well as simulate directly on the platform. For convenience, the model SBML file is provided as File S1 (Data Sheet 1) in Supplementary Material.

### Model Simulations

The Cell Collective platform was used to perform all computational simulations of the model. Although the model is built by using discrete mathematics the output activity levels (AL) can be continuous (ranging from 0 to 100) as previously described in Helikar et al. (2008) and Helikar and Rogers (2009). Each simulation is synchronous and consists of 800 steps, where the activity level of the measured output component is calculated as the fraction of ones (active states) over the last 300 iterations that describe the network’s steady behavior (Helikar et al., 2008; Helikar and Rogers, 2009).

Let *x_*j*_*(*t_*i*_*) denotes a node’s activity on the *i*th iteration and *j*th simulation where *i* = 1, 2, …, *T* and *j* = 1, 2, …, *N*, total is the simulation out of *N* total simulations. We obtain AL as below.

$$\text{AL}=\frac{1}{300N}\sum_{j=1}^{N}\sum_{i=T-300}^{T}x_{j}(t_{i})$$

The model was simulated and analyzed under four biologically relevant environmental conditions that stimulate cell growth, cell death, quiescence, motility (and randomly generated behaviors), as established and detailed in Helikar et al. (2008). The environmental conditions that stimulate each of these cellular responses were obtained based on Helikar et al. (2008) where the model’s responses were characterized based on 10,000 combinations of randomly generated environmental signals. For example, cell growth behavior is characterized by higher AL of Erk (marker for proliferation) and Akt (marker for anti-apoptosis). Cell motility behavior was characterized by higher AL of Cdc42 and Rac. Quiescence response is considered when the activity level of Akt is medium to low, and proliferation (Erk) and motility (Cdc42 and Rac) are low or inactive (Helikar et al., 2008). Each environmental condition is defined by different combinations of AL of external components (ligands). The activity level ranges of the environmental conditions were further determined by an optimization method whereby 2,000 simulations were run with all external stimuli ranging from 0 to 100 (except for IL1_TNF and Stress that were limited to low AL). Subsequently, environmental activity level combinations that stimulated cell growth, cell death, motility, and quiescence most effectively were selected as the corresponding environmental conditions (Table 1). This is directly analogous to optimization experiments in laboratory studies (e.g., determining the optimal medium and plating conditions of a cell before performing a growth factor titration).

**Table 1:** **Activity level ranges of environmental stimuli for cell death, growth, motility, quiescence, and random environments**.

External	Death	Growth	Motility	Quiescence	Random
Extracellular matrix (ECM)	10–72	26–82	81–99	7–30	0–100
Epidermal growth factor (EGF)	3–15	72–97	29–83	43–56	0–100
Calcium pump (ExtPump)	35–87	24–83	41–92	17–82	0–100
GPCR q ligand (alpha_qL)	13–58	18–78	17–74	4–84	0–100
GPCR i ligand (alpha_iL)	1–4	15–77	30–82	31–83	0–100
GPCR s ligand (alpha_sL)	30–87	24–80	20–77	19–46	0–100
GPCR 12/13 ligand (alpha_1213L)	14–65	18–78	12–77	18–67	0–100
IL1_TNF	4–13	8–15	4–13	2	2
Stress	2–5	2–5	2–5	2–3	2

A wild type (WT) experiment (used as a reference) was conducted under each environmental condition without any perturbations. Subsequently, systematic perturbation experiments were conducted under each condition, whereby each component of the model was constitutively activated (activity stuck at 1; gain-of-function/overexpression) or inactivated (activity stuck at 0; loss-of-function/knockout). Each experiment consisted of randomly selecting 100 combinations of AL of the external stimuli from each condition activity range. (The only exception was the random environmental condition, which was simulated 2,000 times.) Each of the 100 combinations were simulated 30 times (i.e., 30 replicates) to ensure consistency of the dynamics in response to a specific combination of stimuli. These replicates were subjected to a Fligner Killeen test of homogeneity of variances, which confirmed that the measured AL of the network components, were homologous for identical combinations of AL of the environmental stimuli.

### Model Analysis

The Kolmogorov–Smirnov (KS) test (Wang et al., 2003) was used to compare the WT dynamics (under each environmental condition) with the dynamics of each perturbation experiment. If the KS test resulted in a *p*-value <0.05, then it has a difference value (DV) equal to the test statistic; otherwise, the DV for a component is 0. Because we are looking at how a node’s perturbation affects the rest of the network, its DV when it is the perturbed node is set to 0.

### Most and Least Influential Components

The most influential components are defined as components that induce the largest changes in the network under a given perturbation. The ranking of the perturbations is derived by calculating an influence score (IS) for the *i*th node, which is found by summing the DV for all *M* nodes in the network. The top 10% are considered most influential, and the bottom components with IS value 0 were considered the least influential. The cutoffs were set to 10% because only a few components had a high influence on the network.

$$\begin{array}{l} {\text{IS}}_{i}=\sum_{j=1}^{M}{\text{DV}}_{\mathrm{ij}}\\ i=1,\;\ldots ,\;130 \end{array}$$

### Most Affected Components to a Specific Perturbation

For each perturbation induced, the components that are most sensitive to that perturbation are ranked in decreasing order to be able to characterize downstream effects of the perturbation on the network.

### Annotation and Biological Relevance of Signal Transduction Components

All model components were first annotated using the appropriate NCBI gene IDs (Pruitt et al., 2007) for associated genes and UniProt IDs (Consortium, 2011) for protein products of the genes. All components were then further characterized using online resources such as DrugBank (Wishart et al., 2006).

The biological process enrichment analysis of the most influential components was done using DAVID (Huang et al., 2008), with high stringency. Gene Ontology (Ashburner et al., 2000), SP_PIR keywords, and KEGG pathways (Kanehisa, 2002) were obtained using FDR < 5%.

Essentiality data were obtained from the Online GEne Essentiality (OGEE) database and mapped on the most and least influential components (Chen et al., 2012). DrugBank data were used to obtain druggability information for each component in the network. Data on cancer-associated genes were obtained from The Cancer Genome Atlas (TCGA) (Weinstein et al., 2013) and mapped on the most influential components to identify cancer-associated most influential components. The enrichment of essential genes and druggable proteins was computed based on the number of genes mapped on most or least influential components out of the total number of most and least influential components.

### Network Motif Analysis

Network motif analysis in the directed signal transduction network was performed using FANMOD tool (Wernicke and Rasche, 2006). The default parameters were used that include 100,000 samples to determine the sub-graphs. The significance of network was computed by comparison with 1,000 random networks. Network motifs that have occurrence more than five times and *p*-value <0.05 were considered as significant. Network motif analysis was previously integrated with logical modeling of signal transduction of epithelial–mesenchymal transition (Steinway et al., 2014).

### Gene Expression Analysis

To investigate the functional activity of the components of signal transduction model, we queried publicly available gene expression data in four different databases (Consortium, 2011); Bgee (gives activity level of genes across different species as well as different developmental stages) (Bastian et al., 2008), CleanEx database (Providing heterogenous data from different) (Praz et al., 2004), Expression Atlas database (gene expression data under different biological conditions) (Petryszak et al., 2014), and GeneVisible database (Gene expression in different tissues) (Zimmermann et al., 2004). Out of 109 signal transduction components i.e., proteins, 107 (~98%) showed expression across different species, developmental stages, organs, and tissues – suggesting the biological activity of signal transduction network. Gene expression status of signal transduction components is shown in the Table S1 in Supplementary Material.

The gene expression dataset GSE53309 was obtained from the GEO database (Barrett et al., 2005; Rosich et al., 2014). We selected samples that were treated with pan-PI3K inhibitor and of normal control. The log 2 RMA signal intensities of samples were transformed into *Z*-scores (Cheadle et al., 2003). To compare the *Z*-scores of treated samples with normal control, we used *Z*-ratio approach. Genes with *Z*-ratio ≥1.50 were considered upregulated and with ≤−1.50 were considered as downregulated. The *Z*-ratio cut-off (1.5) is previously found as robust (Cheadle et al., 2003). The genes of signal transduction components whose AL were affected as a result of PI3K inactivation were examined for *Z*-ratios in both the biological replicates. We used DAVID to perform biological process enrichment analysis of upregulated and downregulated genes. The high stringency and FDR < 5% were used to select significantly enriched biological processes.

## Results

### System-Wide Perturbation Analysis Reveals Core Components of the Signal Transduction Network

**Figure 1:**
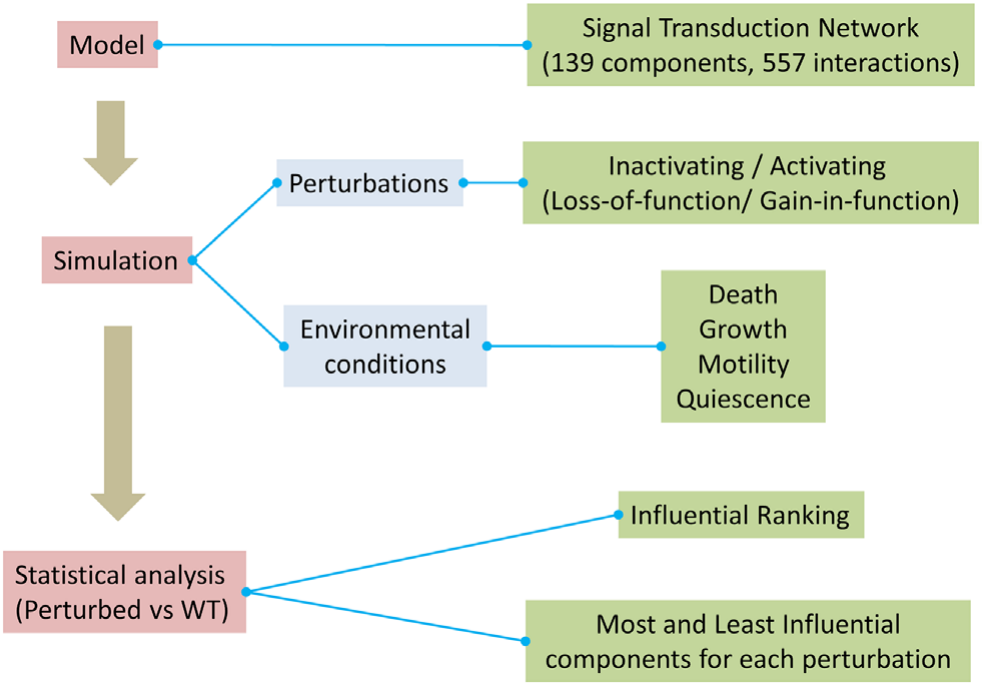
**Overview of the method used to assess influential components in the model**.

A critical objective of biomedical research is the identification and prioritization of novel therapeutic targets. In this context, we performed systematic perturbation analysis in a generic signal transduction model. The workflow used in this work is illustrated in Figure 1.

The activating/inactivating perturbation experiments for each component in the model were carried out across four environmental conditions (as described in the Section “Materials and Methods”). Additional randomly generated extracellular conditions were used to check the robustness of the model and results. Perturbation analysis enabled us to identify and rank components of the signaling network that are most and least influential (Table S2 in Supplementary Material). The heatmaps for all the environmental conditions [Figures S1–S10 (Image 1) in Supplementary Material] indicate that a few components had high influence on rest of the system. Therefore, we considered the top 10% of the components from each condition as the most influential. By contrast, the components that had no influence on the system were considered as the least influential (KS = 0).

**Figure 2:**
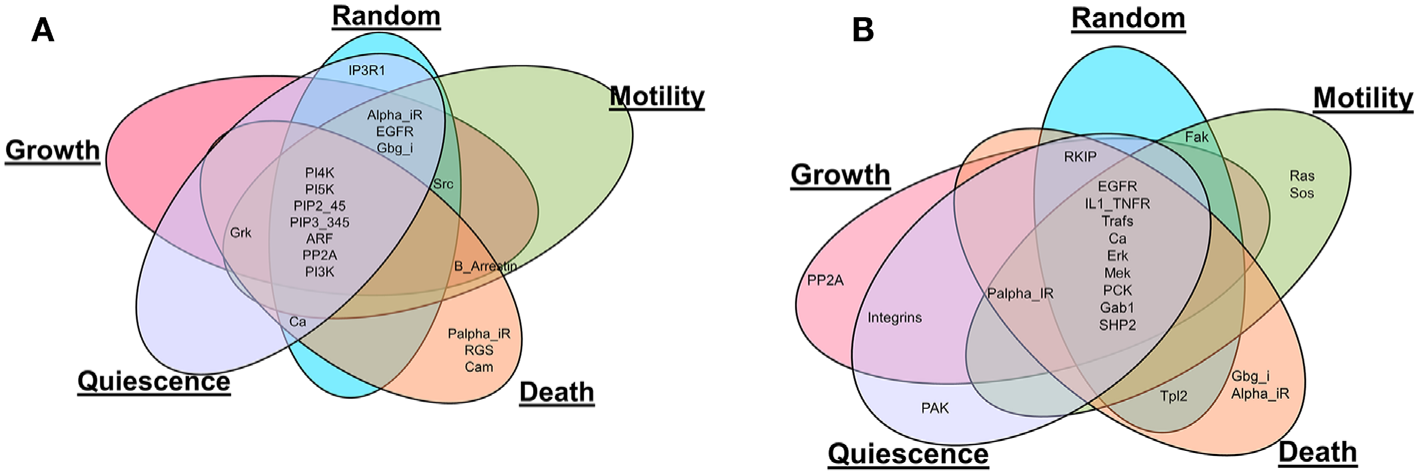
**Comparison of the most influential components across simulated environmental conditions**. **(A)** Inactivating perturbations, **(B)** activating perturbations.

Also, the most influential components correspond to network components that, when perturbed, affect the largest part of the network in terms of the number of affected components and the magnitude of the effect. The most influential components were found for both inactivating (Figure 2A) and activating (Figure 2B) perturbations under the different environmental conditions. It is interesting to note that many of the most influential components overlap across all environmental conditions. However, the most influential components do not overlap between two types of perturbations (inactivating or activating). We investigated whether the most influential components that spanned different environmental conditions could function as housekeeping genes. Also, the most influential components that are specifically found under one environmental condition should have association with that condition.

#### Housekeeping Genes Are Enriched in the Most Influential Components Common in Different Environments

Housekeeping genes are defined as genes expressed at constant level in many cells and under many conditions (Eisenberg and Levanon, 2013). Therefore, components that were identified as most influential under all of the simulated environmental conditions can be hypothesized to have housekeeping function. To investigate this, we compared these most influential components with known housekeeping genes as provided in Eisenberg and Levanon (2013). Under inactivating perturbations, out of the seven components common among the different environmental conditions, PI4K, PI5K, ARF, and PI3K were associated with housekeeping genes (Eisenberg and Levanon, 2013). Under activating perturbations, Trafs, Erk, Mek, and SHP2 (out of nine common components), were associated with housekeeping genes. Housekeeping genes associated with the common components are displayed in Table 2. This observation suggests that the most influential components that are common among different environmental conditions are likely to function as housekeeping genes.

**Table 2:** **Housekeeping genes in the most influential components overlapped among different environmental conditions**.

Perturbation	Components	Genes	Housekeeping genes^a^
Inactivating	PI4K	PI4KA, PI4KB, PIK4CB	PI4KA, PI4KB
	PI5K	PIP5K1A, PIP5K1B, PIP5K1C	PIP5K1A
	ARF	ARFGAP1, ARFGAP2, ARFGAP3	ARFGAP2, ARFGAP3
	PP2A	PPP2CA	PPP2CA
	PI3K	PIK3CA, PIK3CB, PIK3CD, PIK3CG	PIK3C3, PIK3CB
Activating	EGFR	EGFR	No
	IL1_TNFR	IL1B, TNFRSF1A	No
	TRAFS	TRAF1, TRAF2, TRAF3, TRAF4, TRAF5, TRAF6, TRAF7	TRAF6, TRAF7
	ERK	MAPK1 to MAPK15	MAPK1, MAPK6, MAPK8, MAPK9
	MEK	MAP2K1 to MAP2K7	MAP2K1, MAP2K2, MAP2K5
	PKC	PRKCA, PRKCB, PRKCD, PRKCE, PRKCG, PRKCH, PRKCI, PRKCQ, PRKCZ	No
	GAB1	GAB1	No
	SHP2	PTPN11	PTPN11

*^a^List of housekeeping genes were obtained from Eisenberg and Levanon (2013)*.

#### Unique Components Associated with Each Environmental Condition Are Found to Be Condition Specific

Under both types of perturbations, certain environmental conditions had several uniquely associated components (Figure 2; Table 3). Under inactivating perturbations, components uniquely associated with the cell death stimulating condition are calmodulin (CaM), RGS, and Palpha_iR. Out of these, CaM and RGS have been previously associated with cell death and apoptosis (Fisher, 2009; Berchtold and Villalobo, 2014). In fact, CaM plays a central role in the regulation of several cellular functions, including programed cell death (Berchtold and Villalobo, 2014). It is also known that RGS protein can regulate cell death, cell cycle, and cell division (Fisher, 2009). Under activating perturbations, the most influential components associated with the cell death-inducing condition include Gbg_i and Alpha_iR. On the other hand, PP2A was found to be most influential under the growth stimulating condition, Ras and Sos under motility stimulating condition, and PAK under quiescence stimulating condition. These results are also further supported by published studies that reported Gbg_i (GNB) to be involved in mTOR-mediated anti-apoptotic pathways; Gbg_i was also functionally annotated with apoptosis and cell death (Wazir et al., 2013). PP2A was reported as a highly regulated Ser/Thr phosphatase involved in cell growth and signaling (Janssens and Goris, 2001). In pancreatic cancer cell lines, the knockdown of KRAS has been found to lead to the decrease in cell motility and proliferation (Rachagani et al., 2011; Birkeland et al., 2012). Furthermore, the Grb2–Sos1 complex has been found to most likely promote cell motility, and tumerogenesis (Qu et al., 2014). These observations suggest that the proteins, which were uniquely associated with simulated environmental conditions, are most likely to have the association with that condition. Finally, the literature evidence obtained for housekeeping, or condition associated genes, further supports our simulation results.

**Table 3:** **Condition-specific components and literature support**.

Perturbations	Environmental condition	Associated components	Literature
Inactivating	Death	*CaM*, *RGS*, Palpha_iR	CaM- and CaM-dependent signaling systems control vertebrate cell proliferation, programed cell death, and autophagy (Berchtold and Villalobo, 2014). RGS is involved in cell death (Fisher, 2009)
Activating	Death	*Gbg_i* (GNB), Alpha_iR	Gbg_i has been hypothesized to be involved in mTOR-mediated anti-apoptotic pathways. Furthermore, it has been functionally annotated with apoptosis, cell death (Wazir et al., 2013)
	Growth	PP2A	Highly regulated family of Ser/Thr phosphatase implicated in cell growth and signaling (Janssens and Goris, 2001)
	Motility	KRAS, Sos	Knockdown of *KRAS* in pancreatic cancer cell lines leads to decreased motility and proliferation. The Grb2–Sos1 complex may promote cell motility, and tumerogenesis (Qu et al., 2014)

### Key Biological Processes Are Enriched in the Most Influential Components

**Figure 3:**
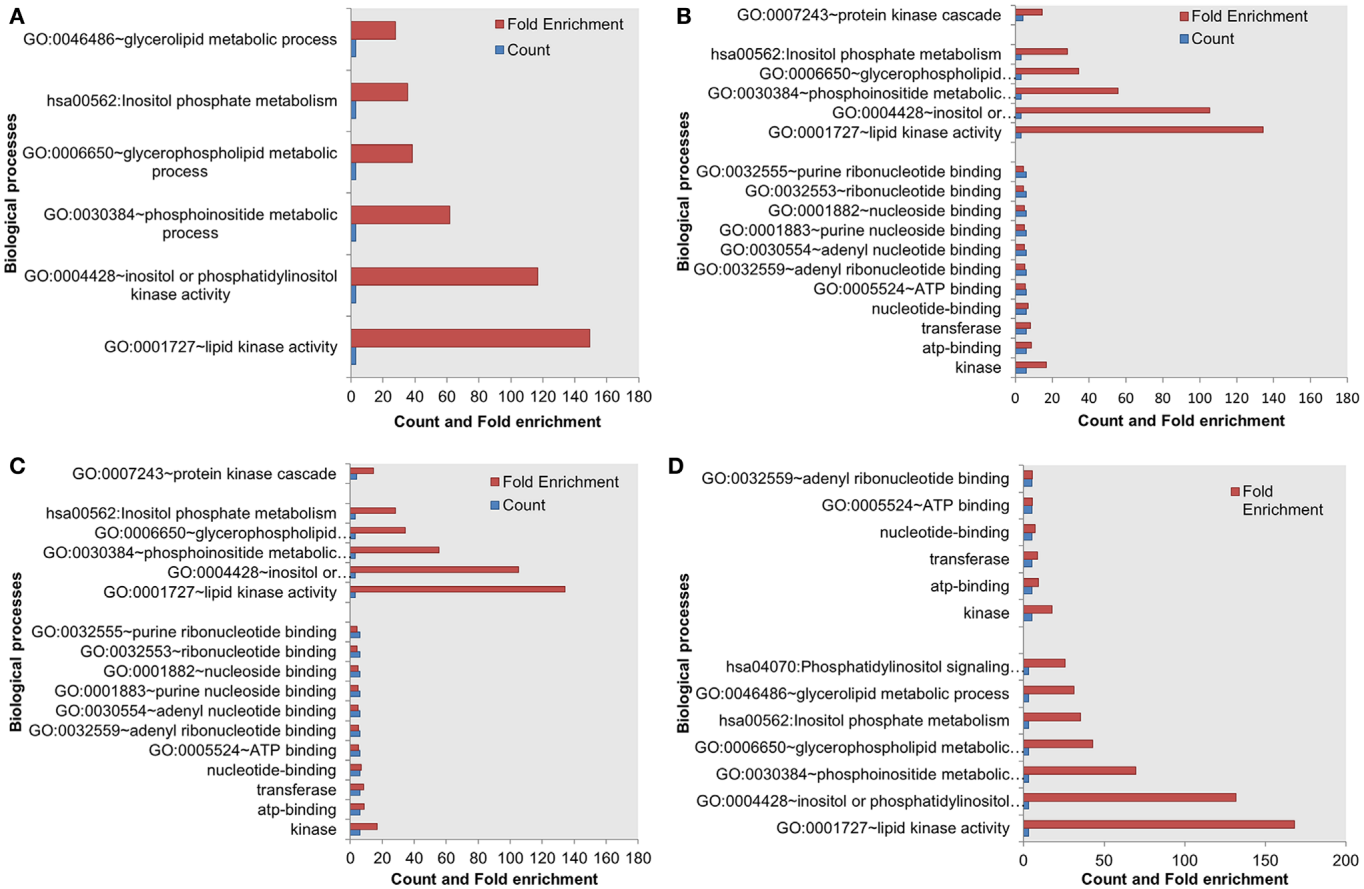
**Enriched biological processes in the most influential components under environmental conditions, and inactivating perturbations**. (A) Death (B) growth (C) motility and (D) quiescence.

**Figure 4:**
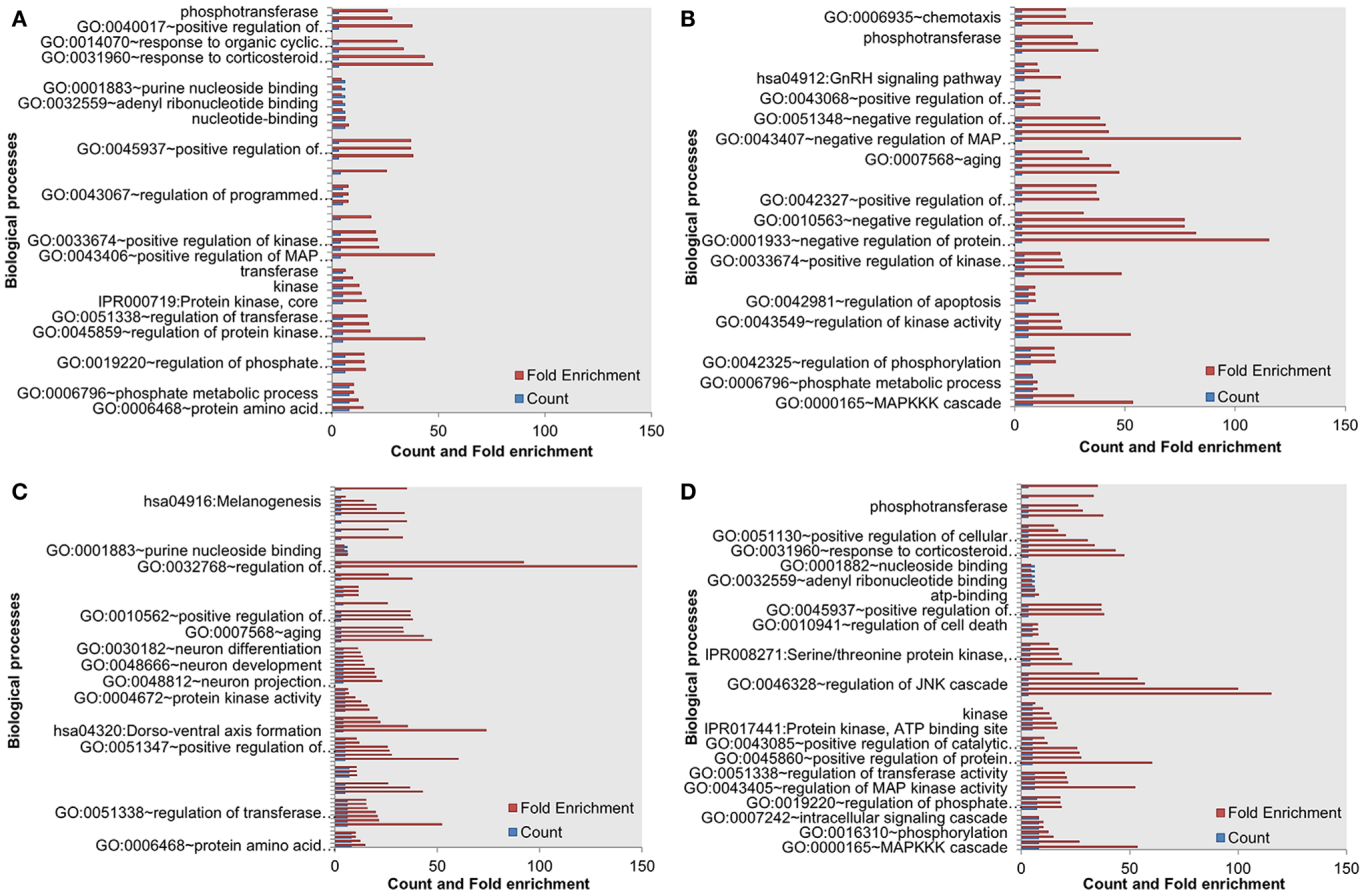
**Enriched biological processes in the most influential components under environmental conditions, and activating perturbations**. (A) Death (B) growth (C) motility and (D) quiescence.

Next, we assessed the enrichment of biological processes or pathways in the most influential components. The most influential components across all four conditions under both types of perturbation showed significant enrichment with key biological processes. The counts and fold differences of enriched biological terms in all the conditions are shown in Figures 3 and 4. In the case of inactivating perturbations, inositol phosphate metabolism was enriched under all environmental conditions (Figure 3). In the case of activating perturbations, the significantly enriched biological processes include phosphate metabolic processes, kinase activity, apoptosis, and, interestingly, the non-small lung cancer pathway (Figure 4). These results illustrate that the group of proteins with similar biological functions appear as the influential components under each type of perturbation.

### The Most Influential Components under Activating Perturbations Are Enriched with Essential Genes

**Figure 5:**
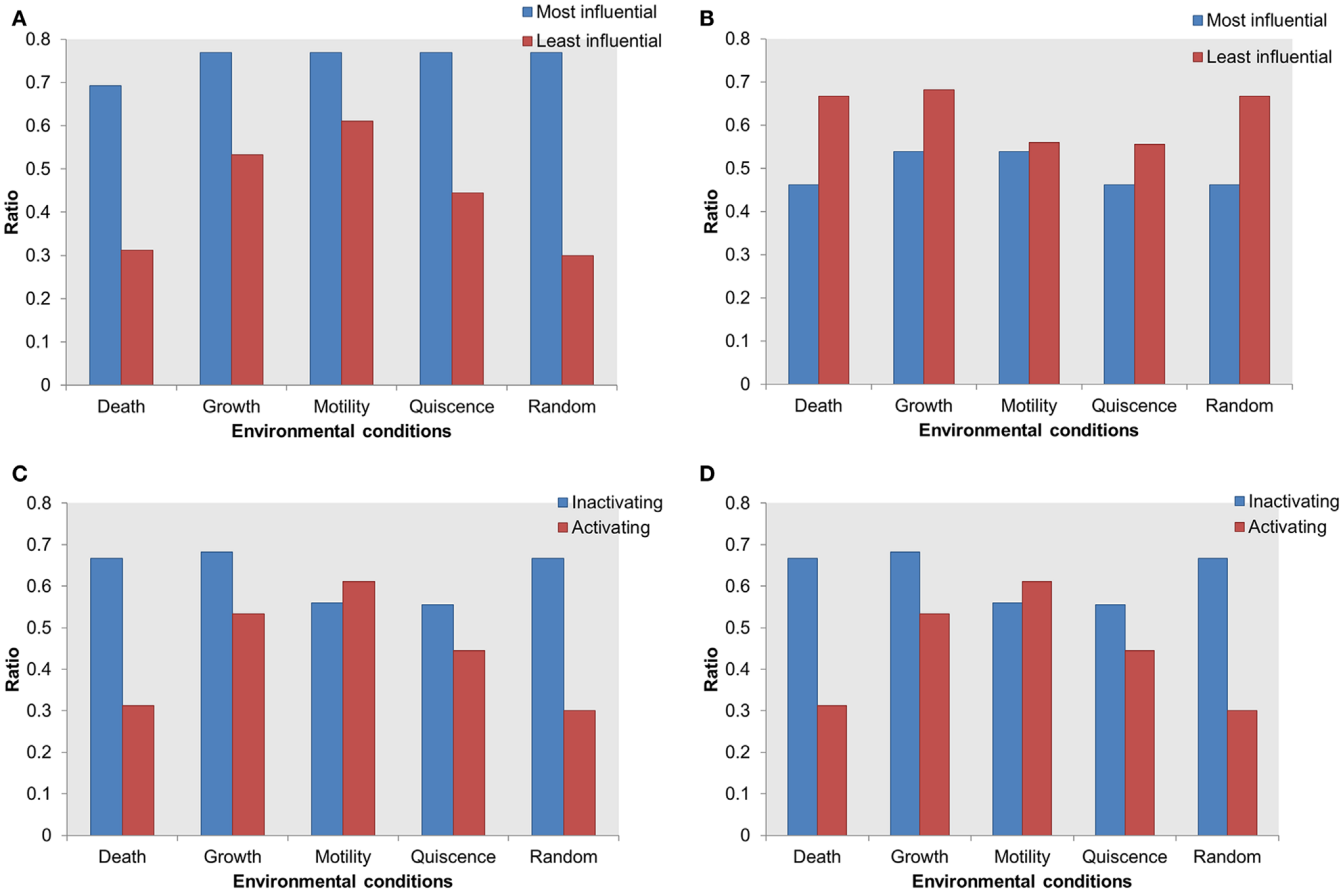
**Distribution of essential genes in the most influential components**. *X*-axis = environmental conditions, *Y*-axis = ratio of essential genes in total selected most or least influential components in **(A)** most influential vs. least influential components under activating perturbations, **(B)** most influential vs. least influential components under inactivating perturbations, **(C)** essential genes in most influential under inactivating vs. activating perturbations, **(D)** essential genes in least influential components under inactivating vs. activating perturbations.

Mutations in an essential gene can be lethal. Based on the hypothesis that the influential components might serve as essential for the survival of the cell, we performed essentiality analysis. We mapped essential genes on the most influential components and on the least influential components. The essential genes mapped on the most influential components were compared with essential genes mapped on the least influential components. Under activating perturbations, more essential genes were found within the most influential components than the least influential components (Figure 5A). Under the cell death stimulating condition, a total of 69% of the most influential components were essential; this is in contrast to the least influential components that contained 31% essential genes. Under other environmental conditions stimulating growth, motility, and quiescence, the difference of essential genes between the most influential and the least influential components are 23, 15, and 32%, respectively.

On the other hand, under inactivating perturbations, we found either an equal or larger number of essential genes in the least influential components (Figure 5B). The most significant differences were observed under the cell death stimulating conditions: the least influential components have 66% of essential genes in contrast to the 46% essential genes in the most influential. Also, under the growth stimulating conditions, 68 and 53% of essential genes were contained within the least and the most influential components, respectively. Under the motility and quiescence stimulating conditions, there were 3 and 9% more essential genes within the least influential components than the most influential components, respectively. We found that under inactivating perturbations, the number of essential genes among the least influential components was slightly larger than the activating perturbation (Figures 5C,D). On the other hand, under activating perturbations, the more essential genes mapped within the most influential components than the least influential components.

Thus, the most influential components are essential under activating perturbations, suggesting an environmental condition-specific essentiality.

### The Most Influential Components Are Enriched with Druggable Proteins

**Figure 6:**
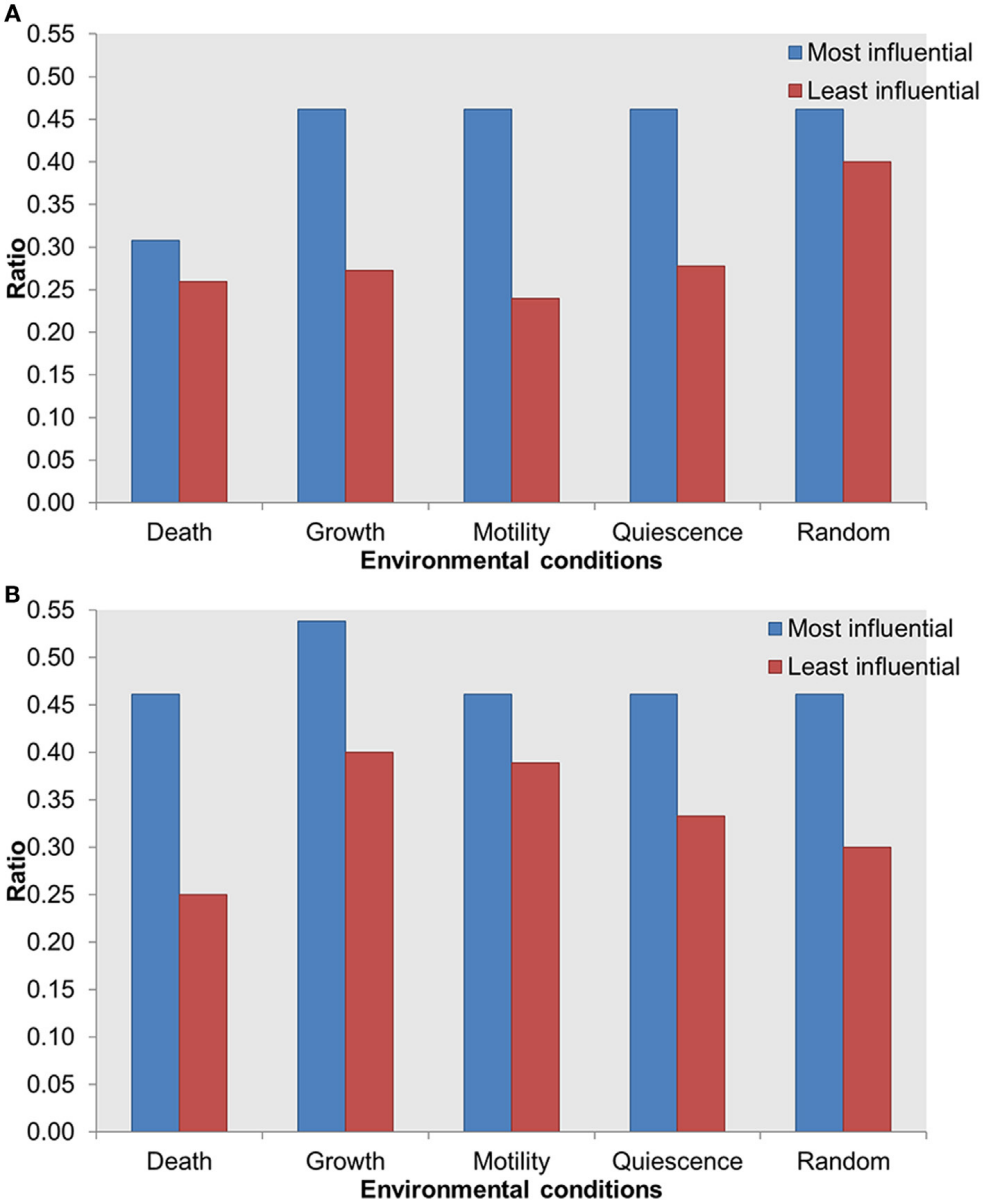
**Distribution of druggable proteins within the most influential vs. least influential components**. **(A)** Inactivating perturbations, **(B)** activating perturbations. *X*-axis = environmental conditions, *Y*-axis = ratio of druggable proteins in total most or least influential components.

To further investigate the importance of the most influential components, we evaluated the distribution of known druggable targets. We obtained druggability data from the DrugBank database (Wishart et al., 2006) and mapped them on the most and least influential components. A total of 51 components in the whole network were enriched with druggable proteins. We compared druggable proteins within the most influential components with druggable proteins within the least influential components. We found that under both types of perturbations and across all environmental conditions more druggable proteins were found among the most influential than the least influential components (Figure 6). Druggable proteins are experimentally characterized or predicted to bind to antagonist or agonist drugs with high affinity. Therefore, enrichment of druggable proteins within the most influential components has the potential to suggest important candidates for therapeutic target discovery.

### The Most Influential Components as Drug Targets

#### Ranked Most Influential Components Based on Downstream Components

**Figure 7:**
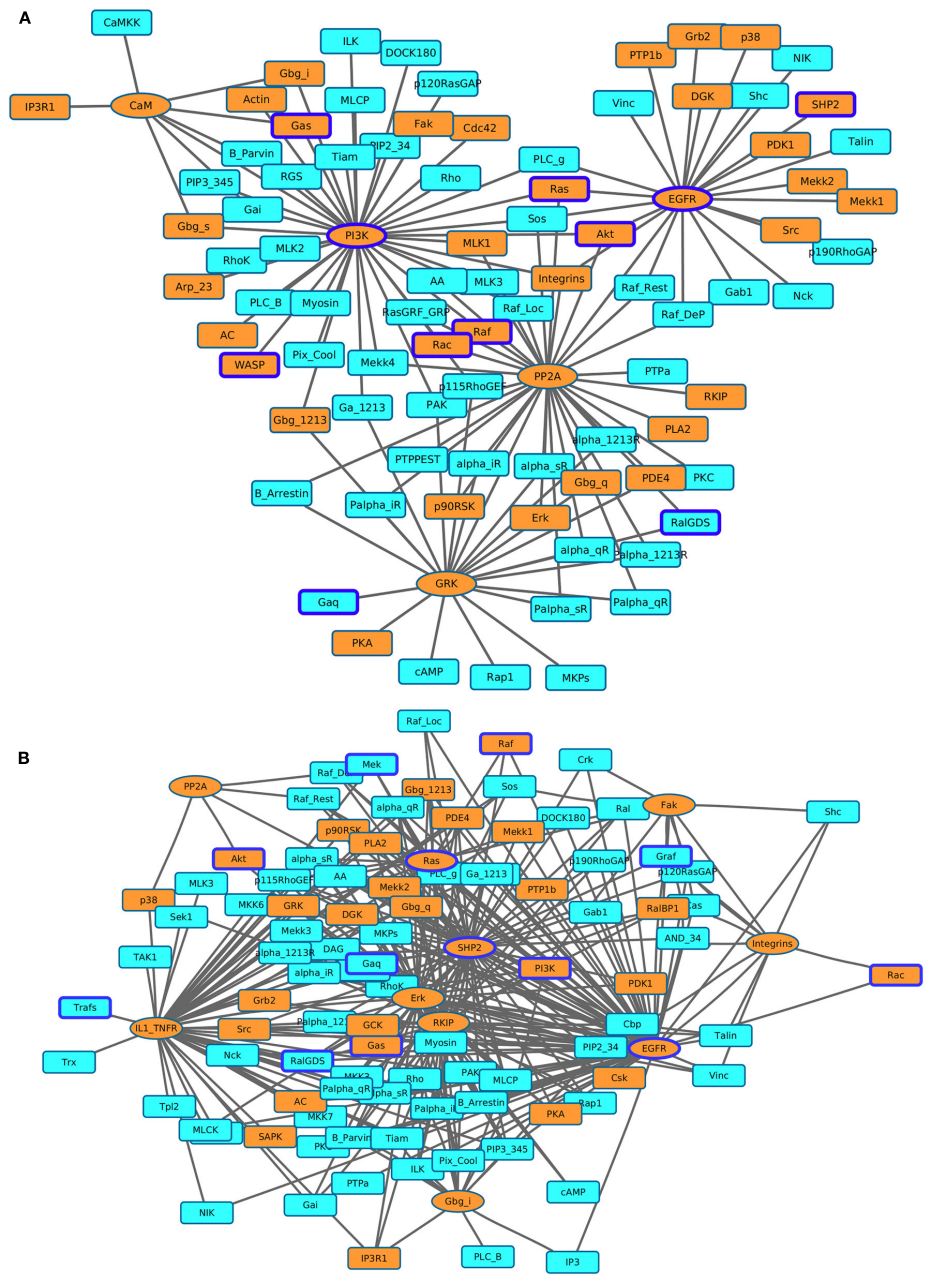
**Visualization of the most affected components (KST value = 1) as a result of perturbing the most influential druggable components**. **(A)** Inactivating perturbations, **(B)** activating perturbations. Orange colored eclipeses = most influential druggable components; squares = affected components; orange colored squares = affected druggable components; components with blue borders = experimentally found to be associated with cancer.

We identified the most affected components of the most influential components under both types of perturbations. We combined all environmental conditions to construct networks of the most influential components with their downstream targets. We subsequently mapped druggable proteins and cancer-associated genes on these networks. Under inactivating perturbations, we obtained a network consisting of the most influential components: PI3K, EGFR, PP2A, GRK, and CaM (Figure 7A). Under activating perturbations, we obtained a network composed of influential components: EGFR, IL1_TNFR, ERK, SHP2, RKIP, Ras, Gbg_i, Fak, Integrins, and PP2A (Figure 7B).

The total number of downstream targets for each of the most influential druggable component under both inactivating and activating perturbations is listed in Table 4. We analyzed if these downstream components also affects their upstream component. In the case of PI3K-out of 42 downstream components, two (PIP3_345 and RGS) are part of a feedback system. Other feedback components in downstream targets include alpha_iR for GRK in inactivating perturbations, Gab1 for SHP2, and Palpha_iR for RKIP under activating perturbations. We observed that EGFR, a validated cancer drug target (Mendelsohn, 2001), affects the largest number of components under activating and inactivating perturbations.

**Table 4:** **Number of downstream targets of the most influential druggable components**.

	Number of affected components	Number of affected druggable components	Number of cancer-associated components	Feedback components	Perturbation
EGFR	70	25	8		Inactivating
EGFR	24	13	3		Activating
IL1_TNFR	54	14	5		Activating
Erk	54	21	8		Activating
SHP2	53	17		1 (Gab1)	Activating
RKIP	43	12	4	1 (Palpha_iR)	Activating
PI3K	42	17	7	2 (PIP3_345, RGS)	Inactivating
PP2A	36	14	6		Inactivating
PP2A	5	3	2		Activating
Ras	30	13	5		Activating
GRK	22	5	2	1 (alpha_iR)	Inactivating
Gbg_i	15	5	1		Activating
Fak	14	6	4		Activating
Integrins	11	3	3		Activating
CaM	8	5	2		Inactivating

#### The Most Influential Components Mainly Affect Other Most Influential Components

**Figure 8:**
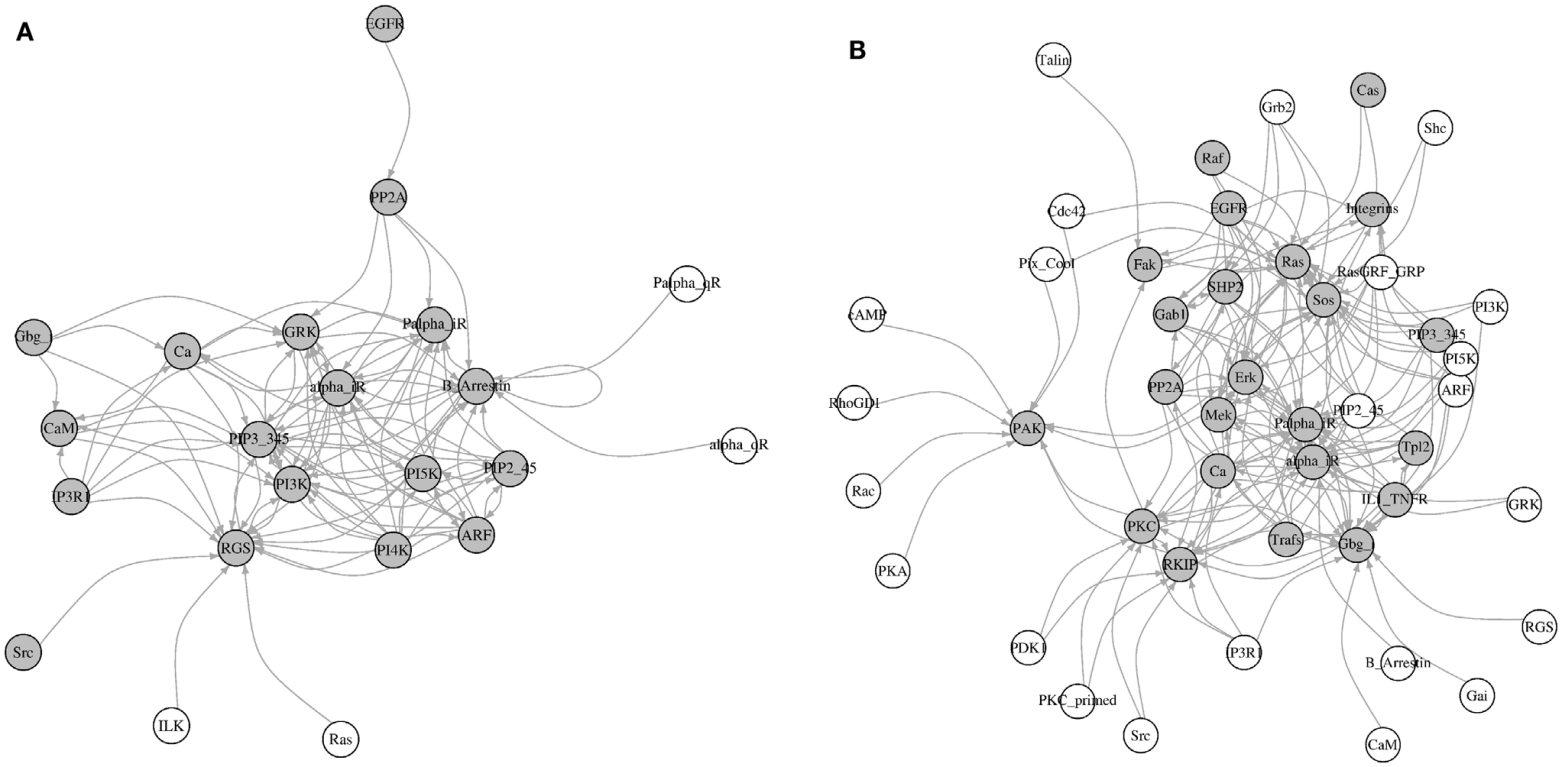
**Visualization of the upstream components affecting the most influential components**. **(A)** Inactivating perturbations, **(B)** activating perturbations. Gray colored nodes = the most influential components, and white colored nodes = not most influential components. The directions of arrows are from the source (upstream component) to the target (most influential components).

Here, we identified all components that directly affect the activity of each most influential component (KS = 1). Interestingly, most of these direct upstream components were also ranked as the most influential in at least one environmental condition (Figure 8). Under inactivating perturbations, 22 components were directly upstream of the most influential components. Of these, 19 were the most influential under at least one environmental condition. On the other hand, under activating perturbations, out of 45 upstream components, 19 were also ranked as most influential. Additionally, under inactivating perturbations, 9 (CaM, EGFR, Gbg_i, GRK, IP3R1, PP2A, PI3K, Ras, and Src) out of total 22 upstream components are druggable. Out of these 22 components, 6 components (CaM, EGFR, Gbg_i, GRK, IP3R1, and PP2A) were upstream to the most influential druggable components. Under activating perturbations, 21 (CaM, Cdc42, EGFR, Erk, Fak, Gbg_i, Grb2, GRK, IL1_TNFR, Integrins, IP3R1, PDK1, PI3K, PKA, PP2A, Rac, Raf, Ras, RKIP, SHP2, and Src) out of 45 upstream to the most influential components are associated with druggable proteins. Out of these 21, 10 components were also the most influential. Under both types of perturbations, a total of 18 (alpha_iR, ARF, B_Arrestin, Ca, CaM, EGFR, Gbg_i, GRK, IP3R1, Palpha_iR, PI5K, PIP2_45, PIP3_345, PP2A, RGS, PI3K, Ras, and Src) upstream components were common. Nine of these components (CaM, EGFR, Gbg_i, GRK, IP3R1, PP2A, PI3K, Ras, and Src) were druggable or these were used as the drug targets. The important drug targets, such as EGFR, PI3K, Ras, and Raf, are also appeared as influential upstream components. Together, these results suggest that under inactivating perturbations the activity of the most influential components are likely to be modulated by the other most influential components.

#### The Most Influential Components as Drug Targets and Drug Resistance

The top most influential components, such as EGFR, PI3K, ERK, and Ras, have been previously explored as drug targets in multiple cancer types. However, it is also evident from literature that several most influential components have been associated with drug resistance. For example, in non-small cell lung cancer, mutation within the kinase domain of EGFR and epithelial–mesenchymal transition are responsible for the development of resistance to gefitinib (Holohan et al., 2013). In colorectal, and head and neck cancers, KRAS mutation, EGFR-S492R mutation, and increased ErBb signaling are responsible for resistance against Cetuximab (Dienstmann et al., 2012; Holohan et al., 2013). Furthermore, PI3K showed drug resistance in breast cancer against rapamycin through the expression of RSK3 and RSK4 (Rodon et al., 2013). Mutations in ERK1 or ERK2 have shown resistance against ERK inhibitors or RAF/MEK inhibitors (Wagle et al., 2014). Tumors with mutation in BRAF V600E can adapt to the RAF inhibitors (Lito et al., 2013; Perna et al., 2015). As such, the identification and prediction of drug targets alone are not sufficient to identify completely useful drug targets. Investigation of the interactions and feedback of these most influential components could be useful to modulate the activity of the most influential component. Thus, we explored the regulatory interactions to investigate the effect of combinatorial perturbations on cell’s behavior.

### Regulatory Interactions between the Most Influential Components and Their Upstream Components

To develop a better strategy that can account for drug resistance of the most important drug targets, we sought to investigate novel regulatory interactions. We analyzed the previously described interactions between the most influential components and their direct upstream components. We found that some interactions consistently occur in more than one environmental condition. For example, the inactivation of IP3R1 increases the activity of PI3K under all four environmental conditions. However, the maximal effect was observed under the death environmental condition. Additionally, the inactivation of IP3R1 leads to inactive RGS under three environmental conditions stimulating cell growth, motility, and quiescence. These finding also correlate with published studies that found that RGS positively regulates apoptosis (Fisher, 2009). Other examples of consistently occurred interactions include: the activation of Grb2 leads to increased AL of Ras under all four environmental conditions, and increased Sos activity under two environmental conditions stimulating death and quiescence. The activation of Rac increases the activation of PAK under environmental conditions stimulating cell death and growth. Overall, we found three types of interactions: inactivation of one component leads to the increase of activity of another component (PI3K–IP3R1, IP3R1–PI3K, and RGS–IP3R1), inactivation of a component leads to decreased activity of another component (IP3R1–RGS), and activation of a component leads to increased activity of another component (Grb2–Ras, Grb2–Sos, and Rac–PAK).

The fold differences of all these interactions are displayed in the Table 5. Under the cell death condition, the inactivation of IP3R1 results in PI3K activity increase by 2.38-fold. Similarly, PI3K inactivation leads to a 5.42-fold increase in IP3R1 activity. In the case of other interactions, the inactivation of IP3R1 leads to inactive RGS under the cell growth, motility, and quiescence stimulating conditions. Under the motility and quiescence stimulating conditions, the inactivation of Gbg_i leads to inactive CaM. The activation of Grb2 increases the activity of Ras 7.40-fold under the cell death stimulating conditions, and 2.13-fold under the quiescence stimulating conditions. Grb2 activation also affects Sos 7.8-fold under the cell death stimulating conditions and 2.18-fold under the quiescence stimulating conditions. An activating perturbation of Rac increases the activity of PAK more than 18-fold under the cell death stimulating conditions, and 5.59-fold under the growth stimulating conditions.

**Table 5:** **Fold differences of the affected most influential component when the upstream component was perturbed**.

Perturbed component	Affected component	Fold differences (perturbed/WT)			
		Death	Growth	Motility	Quiescence
IP3R1 (inactivation)	PI3K	2.38-Fold^a^	1.03-Fold	1.04-Fold	1.14-Fold
PI3K (inactivation)	IP3R1	5.42-Fold^a^	1.18-Fold	1.15-Fold	1.24-Fold
IP3R1 (inactivation)	RGS	NSA	Complete inactivation	Complete inactivation	Complete inactivation
RGS (inactivation)	IP3R1	NSA	1.21-Fold	1.18-Fold	1.24-Fold
Gbg_i (inactivation)	CaM	NSA	NSA	Complete inactivation	Complete inactivation
CaM (inactivation)	Gbg_i	NSA	NSA	1.30-Fold	1.43-Fold
Grb2 (activation)	Ras	7.40-Fold^a^	1.32-Fold	1.39-Fold	2.13-Fold
Ras (activation)	Grb2	0.99-Fold	0.97-Fold	0.99-Fold	1.01-Fold
Grb2 (activation)	Sos	7.87-Fold^a^	1.39-Fold	1.53-Fold	2.18-Fold
Sos (activation)	Grb2	1-Fold	0.97-Fold	0.99-Fold	1.01-Fold
Rac (activation)	PAK	18.41-Fold^a^	5.69-Fold	NSA	NSA
PAK (activation)	Rac	1.18-Fold	1.24-Fold	NSA	NSA

*NSA, not significantly affected (KST value <1)*.

*^a^Twofold or above change*.

To investigate if these interactions are part of any network motifs in the signal transduction network, we performed a network motif analysis. We found that all interactions discussed above were part of network motifs (*p*-value <0.05). IP3R1–PI3K is found in 3 significantly occurred 4-node network motifs and in 15 significantly occurred 5-node network motifs. The other interactions are also found in significantly occurred 4 and 5-node network motifs (Table S3 in Supplementary Material).

These results suggest different types of regulatory effects of activating and inactivating perturbations of direct upstream components of the most influential components.

#### Cotargeting IP3R1 with PI3K

**Figure 9:**
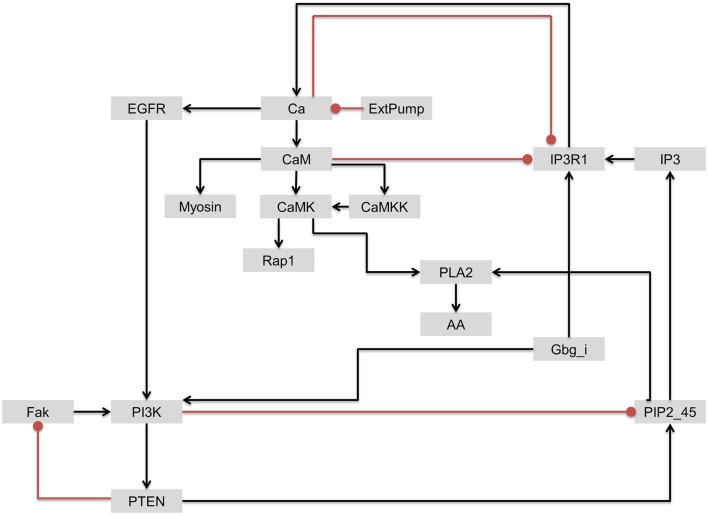
**The regulatory circuit connecting IP3R1 and PI3K and downstream components**. Edges with arrow = activation. Edges with oval end = inhibition.

As discussed earlier, although PI3K was identified as one of the most influential components, it has been also associated with drug resistance. Based on the interactions of upstream regulators of the most influential components discussed above, we further investigated the interactions involving PI3K and IP3R1 with the objective of identifying a secondary drug target that could be potentially used to address the issue of PI3K-associated drug resistance. In contrast to PI3K/Akt signaling, IP3R1 positively regulates apoptosis. We hypothesized that the rate of apoptosis will increase when IP3R1 is overactivated (activating perturbation) and PI3K is inactivated (inactivation perturbation). Despite the strong dynamical relationship between IP3R1 and PI3K, these two components are only connected indirectly through a sub-network. In this sub-network, Gbg_i is upstream of and directly activates both components. IP3R1 regulates PI3K through a Ca → EGFR route, whereas PI3K regulates IP3R1 *via* a PTEN → PIP2_45 → IP3 route (Figure 9).

The inactivating perturbation of PI3K resulted in the inactivation of 29 components across all four environmental conditions. To correlate PI3K inhibition results with laboratory experiments, we analyzed a gene expression dataset obtained from cells treated with PI3K inhibitors (Rosich et al., 2014). In two biological replicates, we found that the genes of components with affected AL had shown differential gene expression (at least in one experiment). As a result of the simulated constitutive inhibition of PI3K in the model, the activity level of a total of 15 components (20 genes) increased more than twofold. Nine (60%) of these components were also significantly upregulated in the gene expression dataset (Table S4 in Supplementary Material). Out of these 20 genes, 9 genes (45%) were upregulated in biological replicate 1, whereas 12 (60%) genes were upregulated in biological replicate 2. Cumulatively, 18 genes (90%) were upregulated in both biological replicates. Two of these signal transduction components, Rap1 and PTPPEST, showed significant upregulation in both the biological replicates in gene expression data. Furthermore, the activity of a total of 26 components (41 genes) decreased more than twofold in our model. Genes of eight components (30%) were significantly downregulated in the obtained gene expression data (Table S4 in Supplementary Material). Out of these 41 genes, three genes (7%) were significantly downregulated in biological replicate 1, whereas eight genes (19.5%) were significantly downregulated in biological replicate 2. Cumulatively, 12 genes (29%) were upregulated in both biological replicates. Furthermore, we compared enriched biological processes within the components affected in the model with enriched biological processes in differentially expressed genes. We found that the “regulation of phosphorylation” biological process was enriched for the upregulated genes in both the model and the gene expression data. For downregulated components, “positive regulation of programed cell death” was consistent for both the model and the gene expression data (biological replicate 1). Together, these results suggest that our simulation results are moderately correlated with the results of available gene expression data. In previous integrative studies of gene expression and biochemical models, at best moderate correlations were observed between gene expression and metabolic fluxes (Blazier and Papin, 2012). Post-transcriptional modifications and enzyme kinetics are possible reasons behind poor correlation between gene expression and protein abundance (Washburn et al., 2003; Blazier and Papin, 2012). As such, more laboratory experiments will be needed to further validate our results.

Under PI3K inactivation, the average activity of IP3R1 increased from 71.9% in WT to 85.18%. This perturbation also led to downregulation of positive regulators of apoptosis phospholipase A2 (PLA2) and arachidonic acid (AA). AA released by PLA2 triggers Ca^2+^-dependent apoptosis through mitochondrial pathways (Penzo et al., 2004). The elevation in Ca^2+^ is thought to be involved in apoptosis (Pinton et al., 2008). It was shown that blocking calcium channels can directly lead to tumor promotion (Mason, 1999). Thus, inactivation of PI3K can block cell proliferation; simultaneously, it can lower the rate of apoptosis. Interestingly, the positive regulation of the programed cell death biological process was enriched in downregulated genes within the analyzed gene expression data.

Under the cell growth stimulating condition, the activating perturbation of IP3R1 increased the activity of apoptosis-associated components: Ca, CaM, CaMK, CaMKK, and RGS in the range of +1.41- to +2.09-fold when compared to WT.

To simulate the cell death effect under the growth stimulating condition, we carried out a double perturbation of IP3R1 and PI3K, whereby IP3R1 was constitutively activated and PI3K was completely inactivated. Under this combinatorial perturbation, we found 27 proteins including proto-oncogenes such as Akt (which suppresses apoptosis) and Raf to be downregulated. Here, we found eight proteins with more than 19% increased activity than in the case of a single inactivating perturbation of PI3K. These proteins include Rap1 (+1.19-fold), Ca (+1.21-fold), CaM (+1.21-fold), CaMKK (+1.21-fold), Myosin (+1.22-fold), CaMK (+1.65-fold), PLA2 (+1.98-fold), and AA (+1.98-fold) (Table 6; full list of all affected components is given in Table S5 in Supplementary Material). These components were downregulated when only PI3K was inactivated. Under the combinatorial perturbation (PI3K inactivated and IP3R1 activated), the increased activity of these components was achieved by constitutive expression of IP3R1 *via* the following routes: IP3R1 → Ca → CaM → CaMK → Rap1 and IP3R1 → Ca → CaM → CaMK → PLA2 → AA (Figure 9). It is noteworthy that these components have been found to positively regulate apoptosis or cell death. Therefore, under the aforementioned combinatorial perturbation, components involved in cell proliferation were downregulated through the inactivation of PI3K, and the activity of tumor-suppressor genes (PLA2) with arachidonic acid (AA) and other components, including Ca, CaM, and CaMK, was increased as a result of the IP3R1 overactivation.

**Table 6:** **Activity of affected components under single (PI3K or IP3R1) and double perturbations (PI3K and IP3R1) under the cell growth environmental condition**.

Affected components	PI3K inactivation (single perturbation)^a^ (fold)	IP3R1 activation (single perturbation)^a^ (fold)	Double perturbation^a^ (fold)	Functional annotation^b^
Rap1	3.25	1.07	3.90	Tumor-suppressor gene
Ca	1.17	1.41	1.43	Calcium ion, apoptosis
CaM	1.17	1.41	1.43	Cell death
CaMKK	1.17	1.41	1.43	Calcium ion binding, apoptosis
Myosin	0.30	1.004	0.36	Regulatory light chain of myosin
CaMK	1.33	2.09	2.19	May function in dendritic spine and synapse formation and neuronal plasticity
PLA2	0.32	1.24	0.63	Tumor-suppressor gene, apoptosis
AA	0.32	1.24	0.63	Apoptosis

*^a^Compared to the activity of components in wild type*.

*^b^Functional annotations for proteins were obtained from UniProt database and literature*.

Together, these results suggest a regulatory interaction between PI3K and IP3R1, and that cotargeting both of these components may serve as therapeutic strategy rather than targeting PI3K alone. Using this combination of targets, we simulated cell death behavior in cell proliferation inducing environmental condition. Thus, we predict that this novel target combination might increase the rate of apoptosis while blocking cell proliferation in tumor cells. However, additional experimental validation is needed to validate this computational result.

## Discussion

We have presented a systemic perturbation analysis of a signal transduction network model to identify and characterize functionally important components. We used these components to explore novel therapeutic strategies against cancer. Specifically, we used a logical modeling approach to analyze the dynamics of a large-scale signal transduction model. Logical modeling approaches have been used, for example, to understand the dynamics of signal transduction and gene regulation networks to identify drug synergies in gastric cancers, and to identify potential drug combinations (Flobak et al., 2015). In biochemical networks, combined effect of topology and dynamical features has been shown to have the most significant impact on the dynamics of the network (Kochi et al., 2014). Computational approaches have become indispensable tools to understand biological pathways and disease phenotypes. Examples include computational methods such as molecular modeling, text mining, and network modeling to identify drug targets in a vast array of diseases from pathogens to complex disorders (Flórez et al., 2010; Yao et al., 2010; Folger et al., 2011; Madrahimov et al., 2013; Puniya et al., 2013).

In the present work, the identified most influential components were characterized for biological functions. The relevance of identified influential components was established with pathway analysis, mapping of housekeeping genes, essential proteins, and association with druggable proteins. Interestingly, we found enrichment of housekeeping genes in the most influential components that were independent of the extracellular environments. A notable agreement is obtained from literature surveys for the most influential components, which were unique to specific environmental conditions. Because essential components are important from a disease perspective, the identified most influential components may serve as potential candidates and essential proteins under specific conditions. Under activating perturbations, we found that essential genes were enriched more within the most influential components than within the least influential components. The high association of dysregulated signal transduction proteins with different subtypes of cancers suggests that these components may be important candidates for drug targets. Notably, the most influential components are enriched with several already known drug targets. However, many of these drug targets (EGFR, ERK, Ras, PI3K, etc.) have been associated with drug resistance (West et al., 2002; Kobayashi et al., 2005; Linardou et al., 2008; Wheeler et al., 2010; Dienstmann et al., 2012). The mechanism of drug resistance includes mutation in the targeted protein or expression of other genes (altered expression) to bypass the effect caused by perturbation, deregulation in apoptosis, etc. (Gottesman, 2002; Holohan et al., 2013). Thus, to identify novel regulatory interactions, we explored components that are upstream to the most influential components associated with drug resistance. Interestingly, several upstream components (more than 90% in the case of inactivating perturbations) to the most influential components were also identified as most influential. Thus, the most influential components form a tightly connected sub-network of proteins interacting with each other. In yeast, it has previously shown that the essential proteins are hubs in the network and have more interconnections than non-essential proteins, and form a module or sub-network (Song and Singh, 2013).

The interaction between IP3R1 and PI3K was observed under all environmental conditions. This interaction was also observed as part of network motifs in the modeled signal transduction network. IP3R1 activation, when combined with PI3K inactivation, increases the activities of PLA2 and AA, which are decreased with a single PI3K knockdown. It was already shown that AA released by PLA2 helps to initiate apoptosis (Penzo et al., 2004). In a *Dictyostelium discoideum* chemotaxis experiment, it was also shown that cells with PI3K deficiency were more sensitive to PLA2 inhibition (Chen et al., 2007), which supports our predicted interaction between PI3K and PLA2. To this end, we hypothesized that the PI3K inactivation could be combined with the overactivation of IP3R1 to increase the activity of proteins involved in apoptosis. IP3R1 inactivation can lead to the downregulation of RGS, and reversibly, the overexpression of IP3R1 can lead to increased activity of RGS. Similar to IP3R1, RGS subtype RGS3T has been found to be involved in inducing cell death (Fisher, 2009), and it has also been found that RGS can suppress the PI3K activity downstream of the receptor (Liang et al., 2009). Therefore, the constitutive activation of IP3R1 might also negatively regulate the activity of PI3K. Systemic analysis of the most influential components and their upstream components has led us to identify novel combinations of drug targets. In various studies, combinatorial therapies have shown a decrease in drug resistance in pathogens. In combinatorial therapy, a protein associated with drug resistance can be targeted in combination with different protein of either the same or different pathway (Fischbach, 2011). Clinical trials have also suggested that the efficiency of cytotoxic drugs increases when given in combinations (Al-Lazikani et al., 2012). If co-occurrence of two genetic events results in cell death, it can be termed as synthetic lethality (Nijman, 2011). The combinatorial perturbation of PI3K and IP3R1 could be considered as synthetically lethal. However, in this perturbation, the activation of IP3R1 is synergistic with the inactivation of PI3K. Upregulation of IP3R1 could be achieved using a targeted drug therapy, such as stress hormone dexamethasone, a synthetic glucocorticoid show to significantly upregulate the expression of IP3R1 in differentiating myoblasts (Chai et al., 2010).

As a validation of model’s result, we used previously published gene expression data. Our model’s results moderately correlate with this data. This agreement was based on only one dataset of PI3K inhibition with two biological replicates. Further addition of experimental data for other perturbations, including the combinatorial perturbation is required to validate the trends of perturbation analysis in model.

In conclusion, by combining IP3R1 (activation) and PI3K (inactivation), we were able to stimulate cell death under the cell growth stimulating condition. Based on this, one can hypothesize that it might be possible that the decrease in cell proliferation with increased apoptosis as a result of this combinatorial intervention could subsequently increase the rate of clearance of tumor cells, and serve as a novel strategy for important targets associated with drug resistance. However, more laboratory validations will be required to test this hypothesis.

## Author Contributions

Conceived and designed the experiments: BP, LA, CH, and TH. Performed experiments: LA and MM. Data analysis and interpretation: BP and CH. Wrote the manuscript: BP, LA, CH, and TH.

## Conflict of Interest Statement

The authors declare that the research was conducted in the absence of any commercial or financial relationships that could be construed as a potential conflict of interest. Tomáš Helikar has served as a scientific advisor and/or consultant to Discovery Collective.

## Supplementary Material

Table S1 **Functional activity of signal transduction components in model**. Gene expression of signal transduction components were queried in four databases: Bgee, CleanEx, Expression Atlas, and Genevisible.

Table S2 **Influential ranking of all the components of signal transduction model**.

Table S3 **Network motifs that containing component pairs selected in Table 5**.

Table S4 **Significant Z-ratios (cells treated with PI3K-inhibitor/untreated) of genes of signal transduction components showed activity level changes in PI3K inactivation in model**.

Table S5 **Activity levels of all the components; on PI3K inactivation (single perturbation), on IP3R1 activation (single perturbation), and on PI3K-IP3R1 (double perturbations)**.

Data Sheet 1 **Model file in SBML format**.

Figures S1–S10 **Heatmaps of raw differences of activity levels between perturbed and WT conditions (for all the simulated environmental conditions under both types of perturbations)**.
