# Supplementary figures and images for: Systems Perturbation Analysis of a Large-Scale Signal Transduction Model Reveals Potentially Influential Candidates for Cancer Therapeutics
Source: Front Bioeng Biotechnol. 2016 Feb 11;4:10. doi: 10.3389/fbioe.2016.00010 (PMC4750464; doi:10.3389/fbioe.2016.00010)

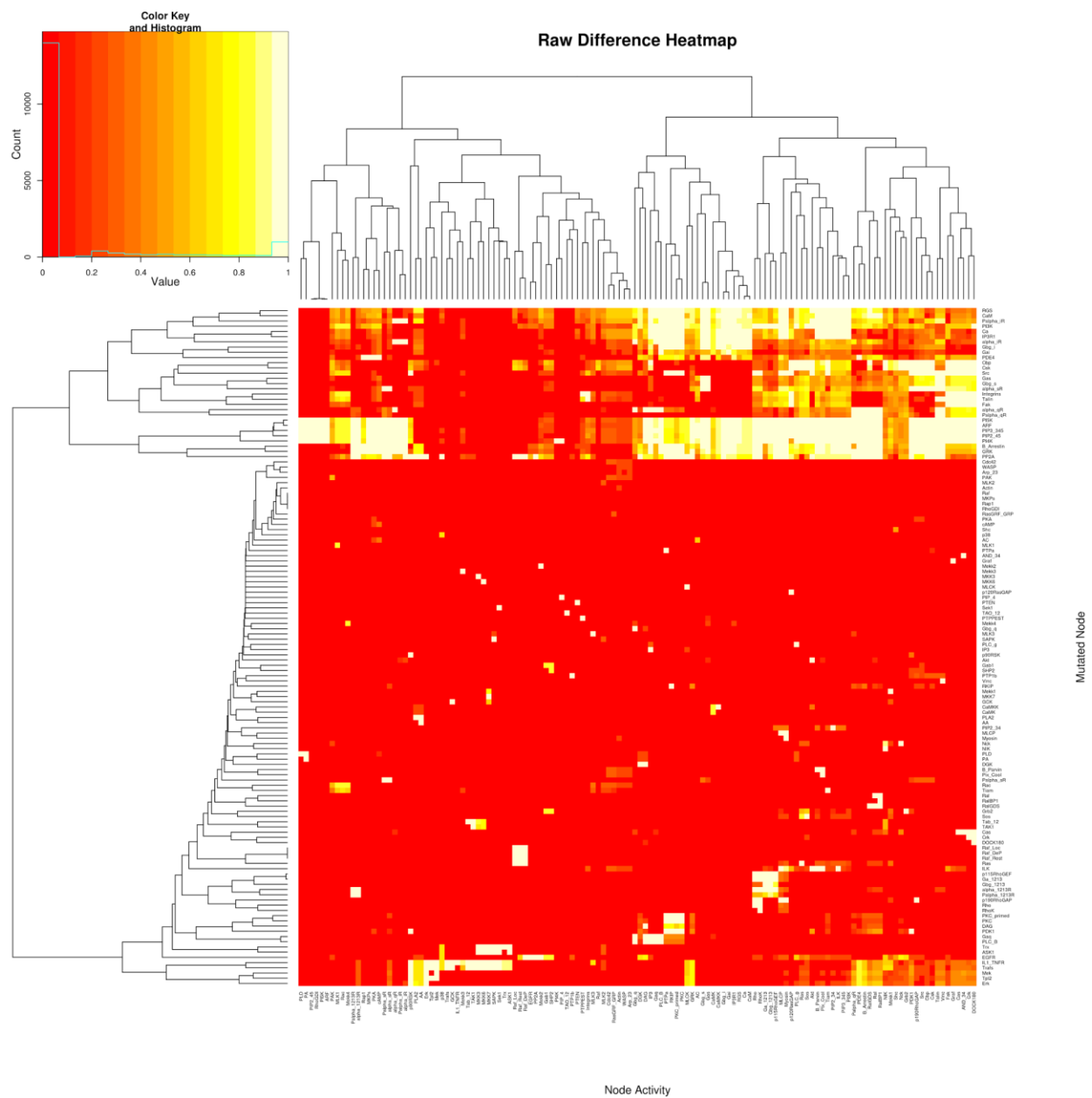

Figure S1: Cell death condition; inactivating perturbations.

Supplement: Supplementary file 7 [file Figures_S1.pdf]

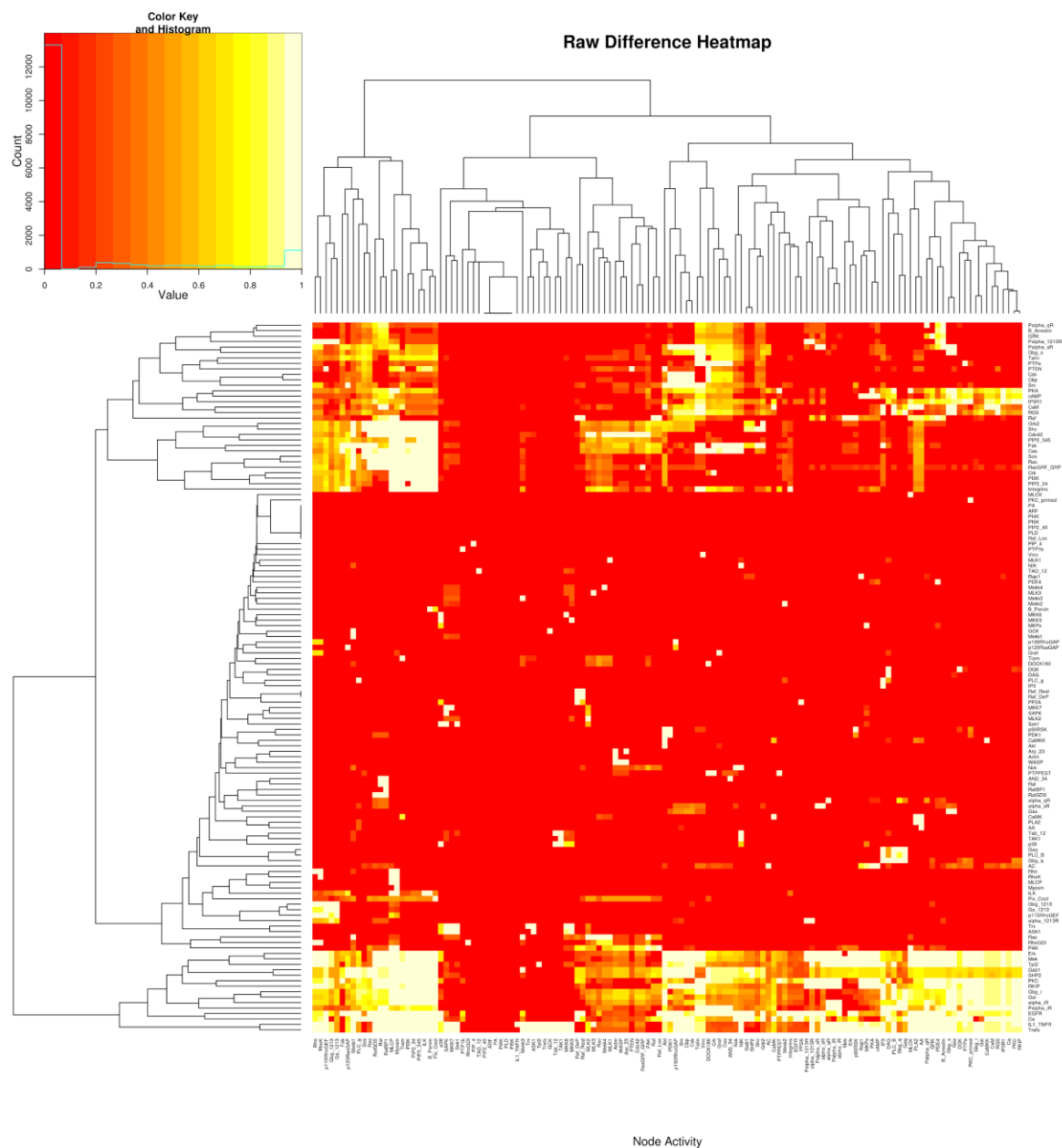

Figure S2: Cell death condition; activating perturbations.

Supplement: Supplementary file 8 [file Figures_S2.pdf]

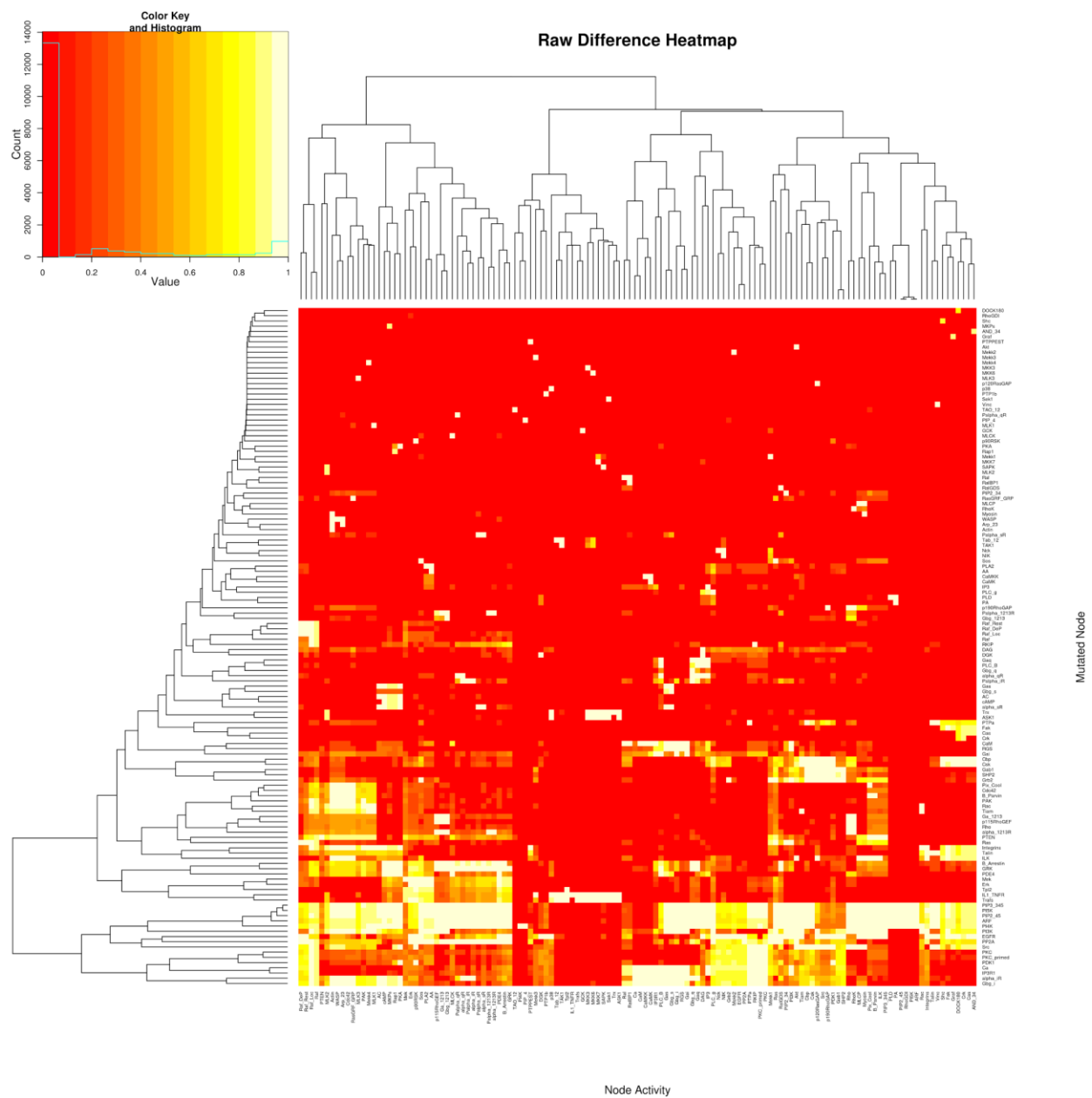

Figure S3: Cell growth condition; inactivating perturbations.

Supplement: Supplementary file 9 [file Figures_S3.pdf]

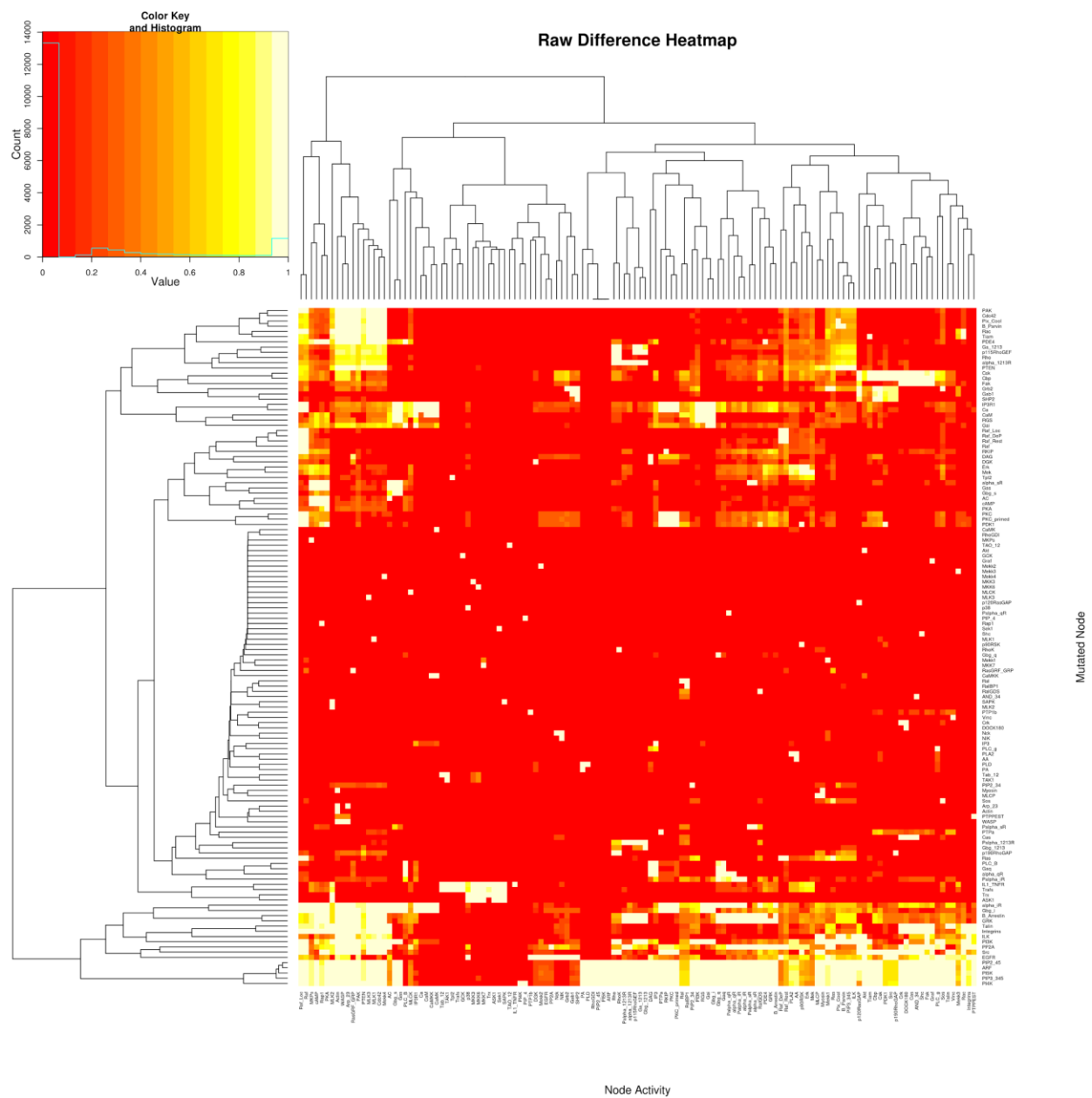

Figure S5: Motility condition; activating perturbations.

Supplement: Supplementary file 11 [file Figures_S5.pdf]

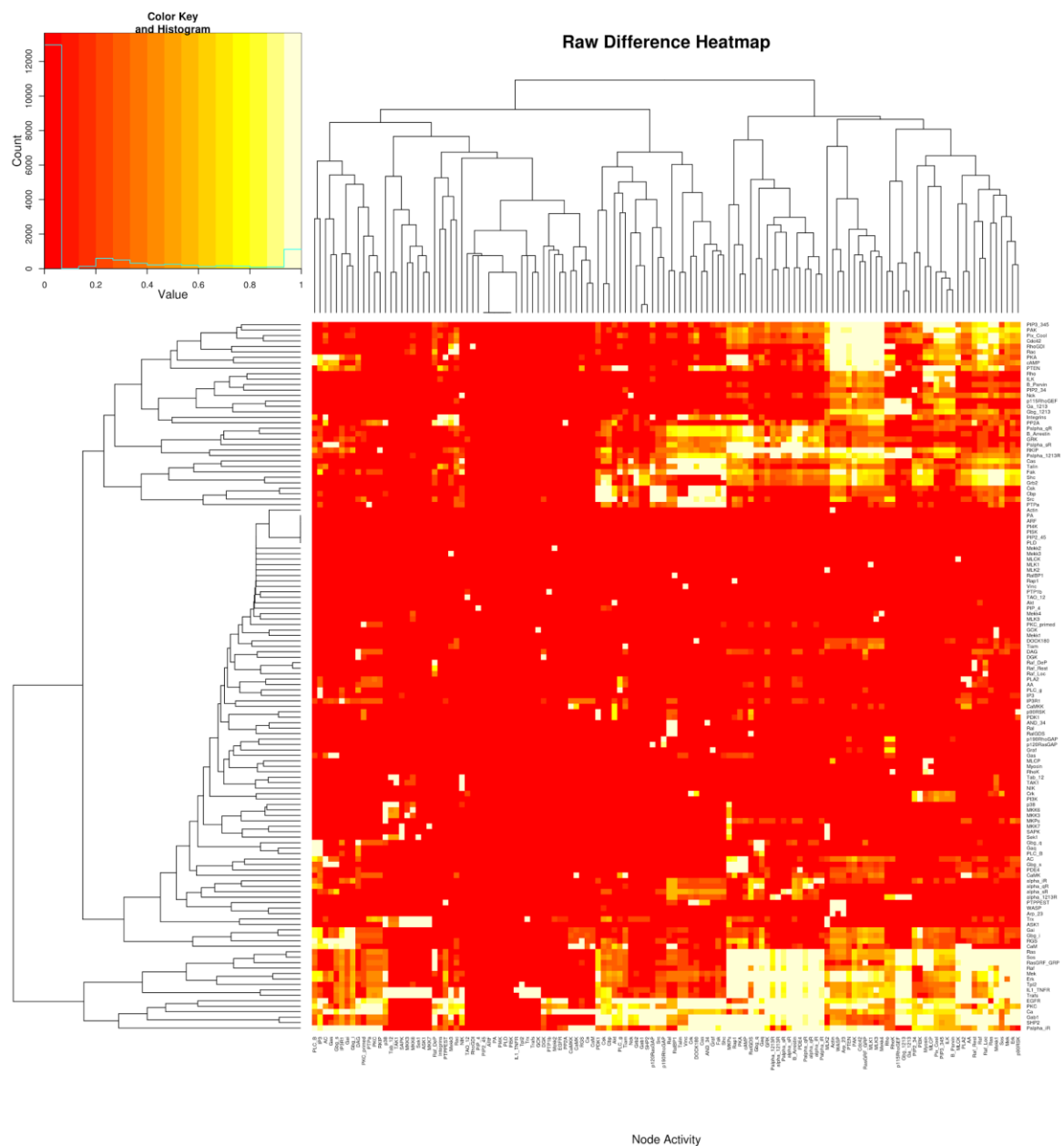

Figure S6: Motility condition; inactivating perturbations.

Supplement: Supplementary file 12 [file Figures_S6.pdf]

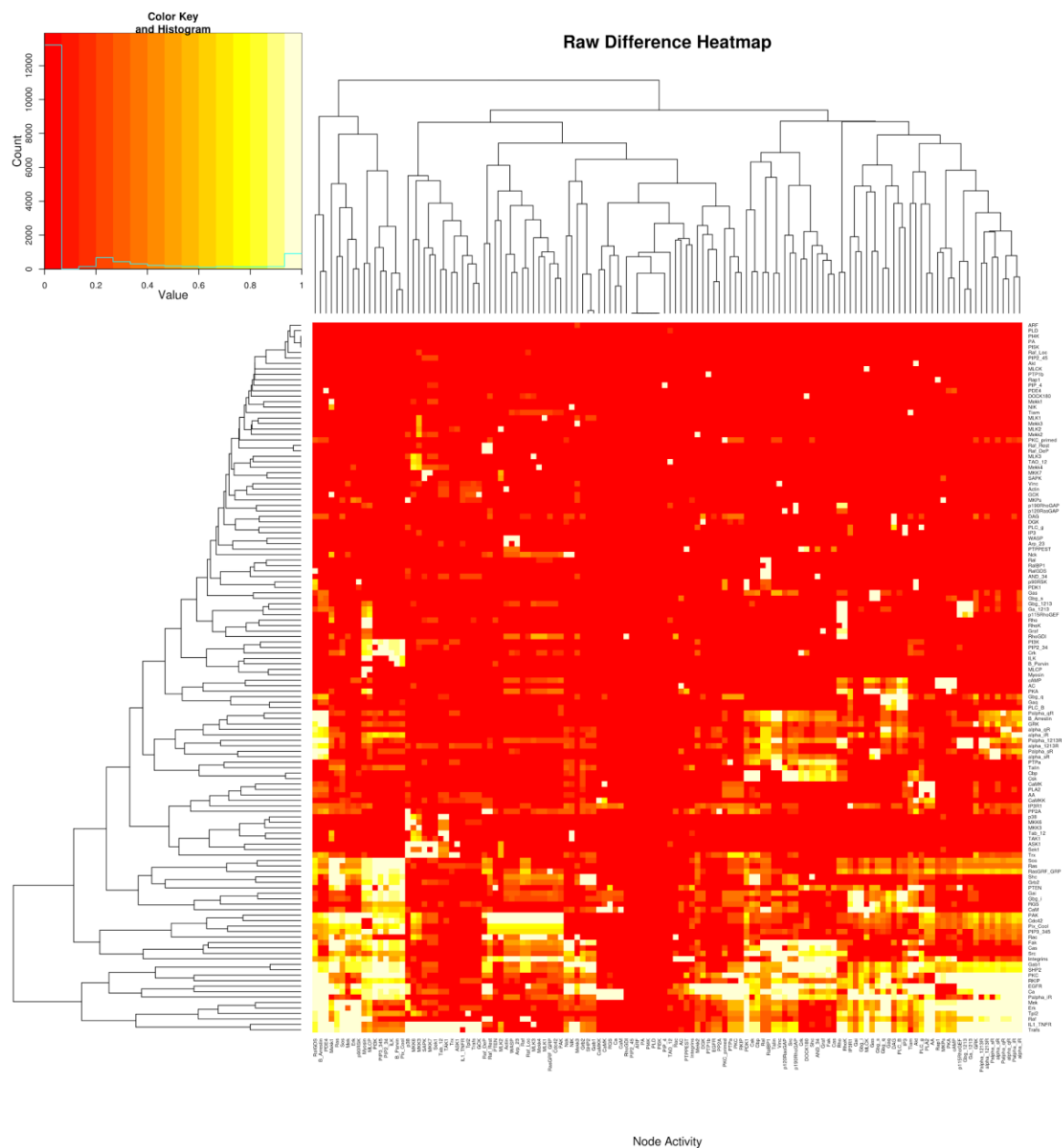

Figure S8: Quiescence condition; activating perturbations.

Supplement: Supplementary file 14 [file Figures_S8.pdf]

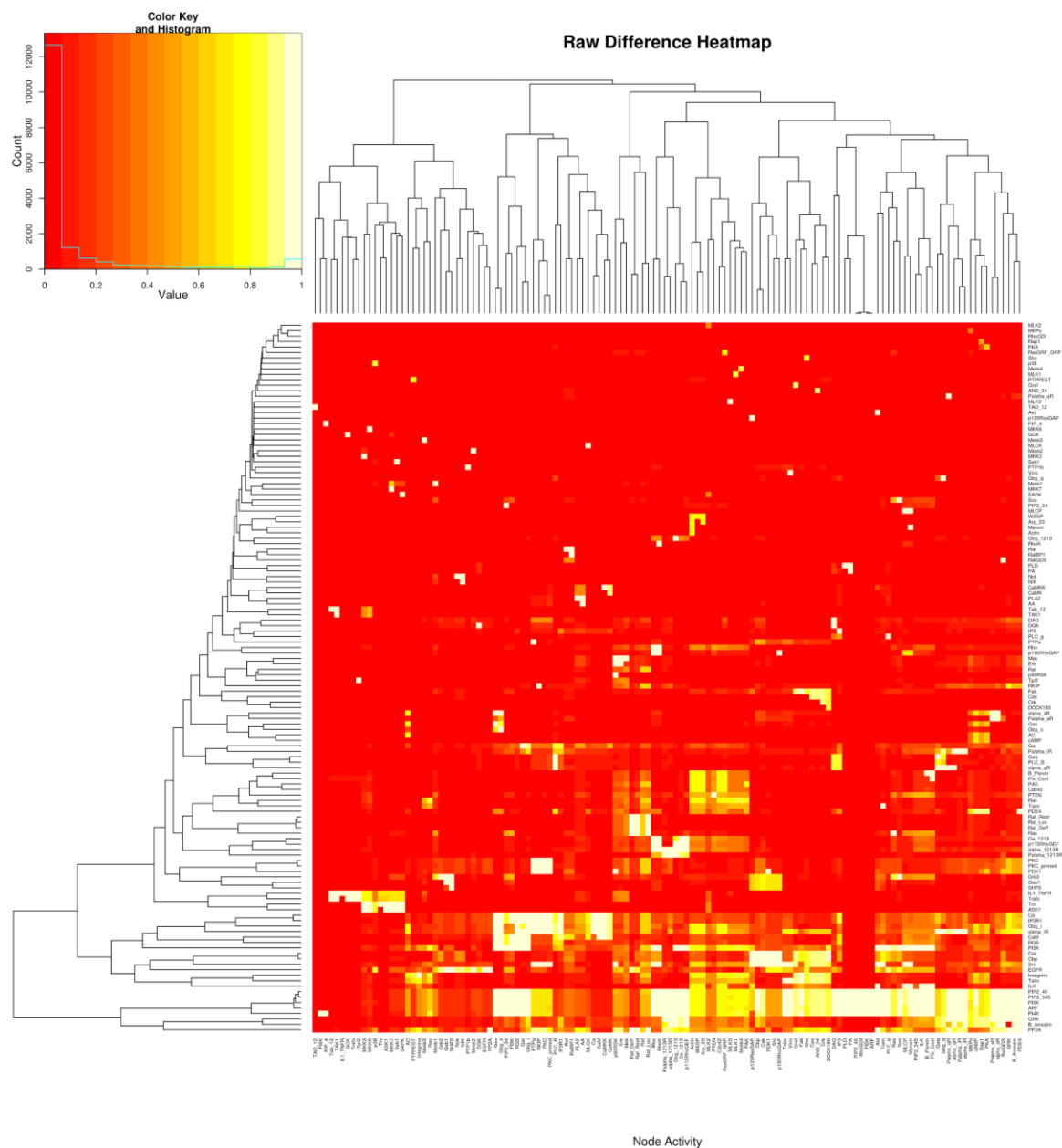

Figure S9: Random condition; inactivating perturbations.

Supplement: Supplementary file 15 [file Figures_S9.pdf]

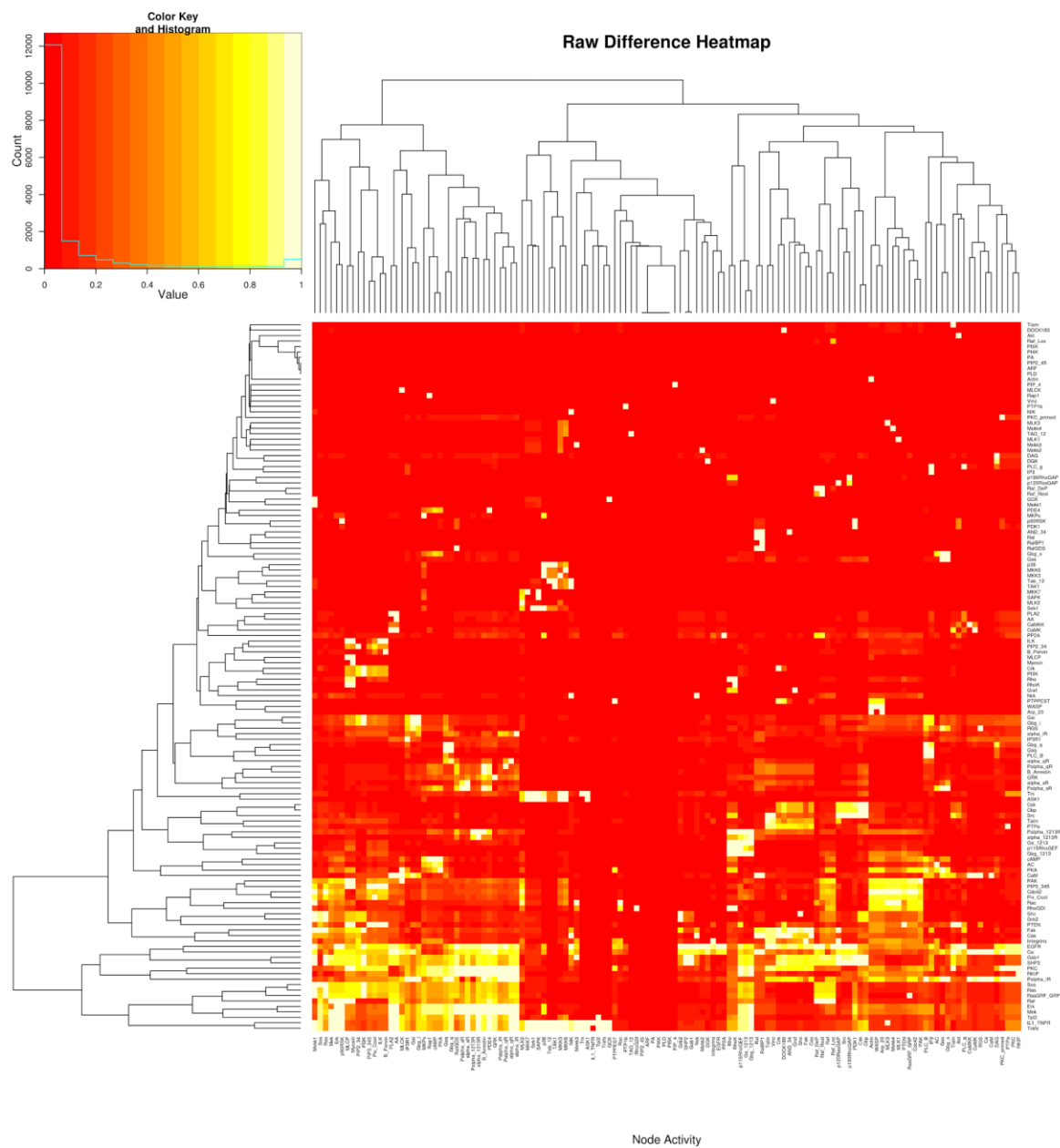

Figure S10: Random condition; activating perturbations.

Supplement: Figures S1–S10 — Heatmaps of raw differences of activity levels between perturbed and WT conditions (for all the simulated environmental conditions under both types of perturbations). [file Figures_S10.pdf]
