# Supplementary material for: Systems Perturbation Analysis of a Large-Scale Signal Transduction Model Reveals Potentially Influential Candidates for Cancer Therapeutics
Source: Front Bioeng Biotechnol. 2016 Feb 11;4:10. doi: 10.3389/fbioe.2016.00010 (PMC4750464; doi:10.3389/fbioe.2016.00010)
Supplement: Supplementary file 10 [file Figures_S4.pdf]

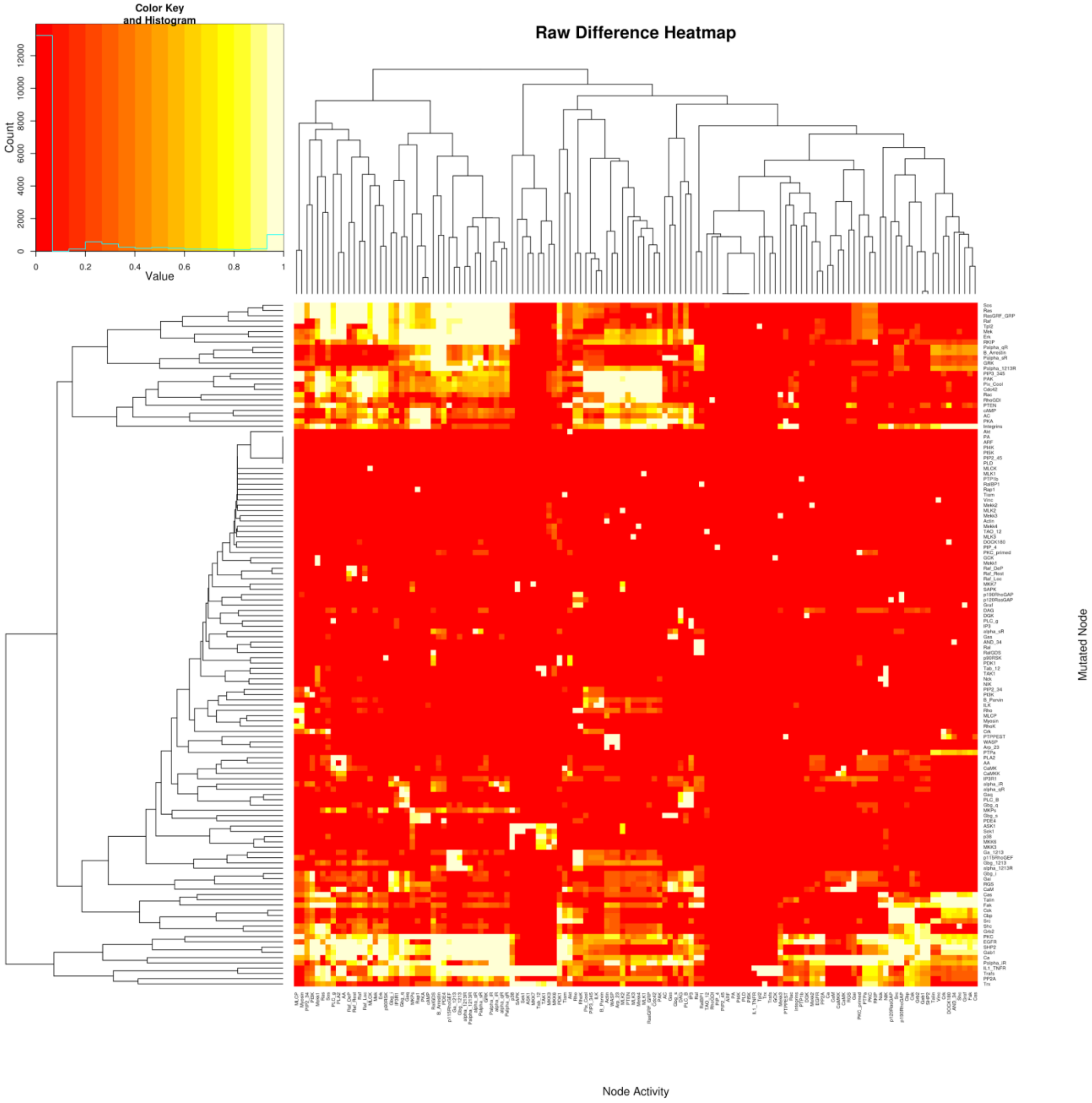

Figure S4: Cell growth condition; activating perturbations.
